# Long-term outcomes of depression up to 10-years after stroke in the South London Stroke Register: a population-based study

**DOI:** 10.1016/j.lanepe.2025.101324

**Published:** 2025-05-15

**Authors:** Lu Liu, Iain J. Marshall, Xianqi Li, Ajay Bhalla, Lidan Liu, Ruonan Pei, Charles D.A. Wolfe, Matthew D.L. O'Connell, Yanzhong Wang

**Affiliations:** a School of Life Course and Population Sciences, King's College London, London, United Kingdom; b NIHR Applied Research Collaboration (ARC) South London, London, United Kingdom; c Department of Ageing Health and Stroke, Guy's and St Thomas' National Health Service Foundation Trust and King's College London, United Kingdom

**Keywords:** Stroke, Cerebrovascular disease, Depression, Mental health

## Abstract

**Background:**

Current evidence on the long-term outcomes of post-stroke depression (PSD) is limited, with most studies relying on short follow-ups and cross-sectional designs. We aim to examine (1) associations between depression at 3-months and long-term outcomes-including mortality, stroke recurrence, functional ability and quality of life (QoL)- up to 10-years; (2) the impact of depression recovery and timing of onset on these associations.

**Methods:**

Data were from the South London Stroke Register (1-January-1997–20-April-2023). Depression was defined as a score >7 on the Hospital Anxiety and Depression Scale. Physical disability was measured using Barthel Index; instrumental activity of daily living (IADL) using the Frenchay Activities Index; and QoL using the Short Form-12, which provides physical and mental health summary scores. Outcomes were assessed annually up to 10-years. Cox proportional hazards models estimated the associations between PSD and mortality and stroke recurrence, while generalized estimating equation was used for physical disability and IADL and linear mixed models for QoL, adjusting for covariates.

**Findings:**

Among 2581 stroke survivors assessed at 3-months, 918 (35.6%) exhibited depression symptom. PSD at 3-month was associated with higher mortality risk (aHR 1.18, 95% CI [1.03–1.36]), but not with stroke recurrence (0.85 [0.64–1.14]) over a 10-year follow-up. The number of patients in analysing the association with physical disability, IADL and QoL was 1388, 1167, and 1292 respectively. PSD was also linked to increased odds of physical disability (aOR 2.94, 95% CI [2.12–4.09]), IADL impairment (2.89 [2.13–3.92]) and lower physical (β = −5.93, 95% CI [−7.26 to −4.60]) and mental QoL (−7.56 [−8.99 to −6.13]) scores. Compared to patients with PSD at both 3-months and 1-year, those recovered by 1-year had similar mortality risk (0.95 [0.76–1.16]), but lower stroke recurrence (0.47 [0.25–0.92]), lower occurrence of physical disability (0.55 [0.36–0.85]) and IADL impairment (0.56 [0.36–0.89]), and improved physical (3.55 [1.30–5.80]) and mental (10.91 [8.56–13.25]) QoL. PSD at 1-year or 5-years was also associated with increased mortality (1-year: 1.33 [1.15–1.53], 5-year: 1.37 [1.10–1.71]), increased risks of physical disability (1-year: 2.20 [1.77–2.74], 5-year: 2.42 [1.39–4.22]) and IADL impairment (1-year: 3.00 [2.22–4.06]; 5-year: 2.69 [1.76–4.11]) and lower physical (1-year: −6.49 [−7.60 to −5.38]; 5-year: −6.78 [−8.30 to −1.24]) and mental QoL (1-year: −12.04 [−13.25 to −10.83]; 5-year: −6.76 [−8.81 to −4.72]) scores.

**Interpretation:**

PSD had lasting impact on stroke recovery, extending significantly beyond the acute phase. As recovery from depression within 1-year is associated with improved health outcomes, further research is needed to develop effective PSD interventions and enhance long-term stroke prognosis.

**Funding:**

10.13039/501100000272National Institute for Health and Care Research (NIHR202339).

**Research in context**

**Evidence before this study**

Post-stroke depression (PSD) is associated with increased mortality and poorer functional outcomes, but most of the previous studies have short follow-ups within 1-year after stroke. A comprehensive literature search was conducted from database inception to 31 March 2025 across Medline, Embase, PsycINFO, and the Web of Science Core Collection. The search strategy included terms such as “stroke”, “post-stroke”, “depression”, “depressive disorder”, “outcomes”, “mortality”, “survival rates”, “stroke recurrence”, “functional disability” “physical disability” and “quality of life” to identify relevant studies. No restrictions were applied regarding language, sample size, or duration of follow-up. The search identified two studies that estimated the natural history of PSD with follow-up periods extending beyond 5 years (specifically, one with a 15-year and another with an 18-year follow-up). However, no studies were found that investigated the association between PSD and adverse health outcomes beyond 5-years post-stroke. This highlights a critical gap in understanding the long-term effects of PSD on stroke recovery and outcomes. The American Heart Association (AHA) emphasizes the importance of timing of PSD assessment in its clinical correlates, but there remains limited research examining how depression at various time-points after stroke correlates with outcomes. Moreover, PSD presents a remitting-relapsing course, it is important to understand whether recovery from depression affects the associations of poor health outcomes. Detailed longitudinal studies are required to further understand the effects of the complex, dynamic nature of PSD on outcomes.

**Added value of this study**

To address these gaps, we conducted a prospective, population-based cohort study, utilizing longitudinal data analysis to estimate^1^ the associations between depression at 3-months and mortality, stroke recurrence, functional outcomes and quality of life (QoL) over a period of up to 10-years after stroke^2^; how recovery from depression and timing of depression onset affect these associations over the long term. We found that PSD is associated with higher mortality, poorer functional outcomes and reduced QoL. These associations of poor health outcomes persisted up to 10-years after stroke, even though the differences in physical disability and QoL declined over time. Recovery from PSD within the first year was associated with lower risks of stroke recurrence, better functional outcomes and improved QoL. Timing of depression symptom onset did not affect the associations of poor health outcomes.

**Implications of all the available evidence**

This research highlights the importance of incorporating ongoing mental health monitoring and support in stroke rehabilitation programs to enhance long-term recovery prospects. Clinicians should recognize the significant impact of PSD on stroke survivors, not only during the immediate post-stroke period but also in the long term, once physical recovery has plateaued. Given that the resolution of depression within 1-year has been shown to be associated with functional outcomes and QoL, it is recommended that targeted interventions and continuous follow-up can be implemented for patients experiencing depression to improve their long-term prognosis. Ongoing screening for PSD should be considered in the long-term, as the onset of depression several years after a stroke continues to be associated with poor health outcomes.

## Introduction

Depression affects approximately one-third of stroke survivors at any time-point.^1^ Post-stroke depression (PSD) has shown to be associated with increased mortality, poorer functional outcomes and reduced quality of life (QoL). However, current studies on the functional outcomes of PSD rely on short follow-ups and cross-sectional designs.^2^, ^3^, ^4^ A review of the literature reveals that no study has explored the association with functional outcomes beyond 5-years employing longitudinal methods to model repeated measurements. This highlights a critical gap in understanding the long-term effects of PSD on stroke recovery and outcomes.

PSD has been characterised as following a remitting-relapsing course with one third of participants remitting without relapsing,^5^ so it is important to understand whether recovery from depression affects the associations of poor health outcomes. Moreover, the mechanism of PSD is multifactorial, and depression at the acute stage following stroke may be pathologically different from depression occurring later after stroke.^6^ Previous studies suggested that the associations with mortality may differ depending on whether depression was measured during the acute phase or chronic phase.^7^ However, less is known about the association of timing of depression onset with functional outcomes.

A previous study by our group in 2014 based on data from South London Stroke Register (SLSR) estimated the effect of recovery from depression and investigated depression as an exposure at more than one time-points (3-months and 5-years).^8^ This study found that depression at 3-months was independently associated with higher mortality and poorer functional outcomes up to 5-years after stroke and recovery from depression did not alter these risks. Depression at 5-years was not associated with poor health outcomes. However, the small sample size limited the statistical power to produce robust results.

The present study extends our earlier analysis by using longitudinal analysis to track changes over time, with a longer follow-up, larger sample size and more comprehensive adjustments. The primary aim is to estimate the association between depression at 3-months after stroke and mortality, stroke recurrence, functional outcomes and QoL up to 10- years after stroke. We further aim to assess the impact of depression recovery and timing of onset on these associations.

## Methods

The SLSR is a an ongoing, prospective, population-based study, which aims to recruit all patients with a first stroke in a defined geographical area of south London, within Lambeth and North Southwark boroughs.^9^ Stroke was defined using the World Health Organization ICD-10 criteria.^10^ Participants are identified by ‘hot pursuit’, where clinical fieldworkers regularly scrutinise records from overlapping sources of notification including hospital stroke units, radiology departments, and relevant out-patient clinics, including GP and A + E referrals to stroke/TIA clinics. Data from patients registered in the SLSR between 1 January 1997 and 31 December 2022 and followed up until 20 April 2023 were used.

### Ethics approval

The SLSR has ethical approval from the NHS Health Research Authority (Wales REC 1 Research Ethics Committee, reference number: 22/WA/0027), and previously from the ethics committees of Guy's and St Thomas' Hospital Trust, King's College Hospital, Queens Square, and Westminster Hospital (London). All patients or their relatives gave written, informed consent to their participation in the study.

### Participants and recruitment

Participants were registered during the acute phase of their first stroke. Baseline data included age, sex, ethnicity (White, Black, Other), socioeconomic status (measured by index of multiple deprivation (IMD), with higher score indicating more deprived), stroke subtype (ischemic or haemorrhagic stroke), smoking (never, ex-smoker, current smoker) and comorbidities. Comorbidities included diabetes, hypertension and heart disease (congestive heart failure, ischemic heart disease, and atrial fibrillation). Physical disability was assessed using the Barthel index (BI)^11^: scores of 0–14 indicated moderate to severe disability, scores ≥15 indicated mild disability. Stroke severity was measured by the National Institutes of Health Stroke Scale (NIHSS): scores >20 indicated severe stroke, scores 5–20 indicated moderate stroke and scores 0–4 indicated mild stroke.^12^

### Follow-up

Follow-up data were collected by face-to-face, postal or telephone instruments with patients and/or their careers, depending on the capacity of patient. After initial assessment, participants underwent follow-up interviews at 3-months, 1-year, then annually after stroke until Mar 2014. Since April 2014 participants have been followed up at 3-months, 1-year, 5-years, 15-years then annually. Patients lost to follow-up at one time-point were contacted again for the following assessment.

#### Depression assessment

Depression symptom was assessed using the Hospital Anxiety and Depression Scale (HADS).^13^ The HADS comprises 7 items each for depression and anxiety, with responses rated on a 0–3 scale. This results in two subscales, the HADS-Depression (HADS-D) and the HADS-Anxiety (HADS-A), each yielding a score range of 0–21. Although HADS is well validated and widely used in stroke patients,^14^ we acknowledge that HADS is not a diagnostic scale but a screening tool that indicates risk of depression, with HADS-D score> 7 indicates possible cases of depression. However, we use the term depression symptom in patients with scores >7 in this paper for succinctness. Patients with scores >7 at 3-months and ≤ 7 at 1-year, were considered recovered. Those with scores >7 at both 3-months and 1-year were categorised as having persistent depression symptoms. Since depression cannot reliably be assessed by proxy, those with severe cognitive or communication problems that the fieldworker judged would give invalid responses were not assessed. For this analysis we used PSD at 3-months as the exposure for the primary analysis. Further analyses assessed relationships with recovery from PSD at 1-year, and with PSD at either 1 or 5-years after stroke.

#### Outcome measurements

Death was confirmed by UK's Office for National Statistics (ONS). Recurrent strokes were identified using the same method as for first strokes and through self-report at follow-ups. Self-reported outcomes were collected yearly post-stroke, with the first 10-years used for this analysis. Functional outcome included physical disability (BI) and Instrumental activities of daily living (IADL) (Frenchay Activities Index: <15 = impaired IADL).^15^ QoL was measured using the Short Form-12, producing 2 summary scores (range from 0 to 100, higher score represents better outcomes) to measure physical and mental health, respectively.^16^ Other data included comorbidities and antidepressants (tricyclic antidepressants, selective serotonin reuptake inhibitors (SSRIs) or others).

### Statistical analysis

χ^2^ and t tests were used to compare demographic and clinical characteristics at 3-months, at 1-year and at 5-years between patients with and without depression symptom and between those assessed and not assessed for depression at each time-point.

Cox regression was used to analyse the association between PSD at 3-months and all-cause mortality up to 10-years after stroke. The proportional hazards assumption for the mortality and recurrence outcomes was assessed using the Schoenfeld residuals, and no violation was observed. Time-to-event was calculated as time from the date of depression assessment. Patients who died before 3-months after stroke were excluded. The follow-up time was up to 10-years with the median of 6.1 years. Mortality follow-up was censored at date of death or 20 April 2023 for surviving participants. A sensitivity analysis excluding patients with pre-stroke depression was performed as its effect on mortality.

Stroke recurrence was censored at date of first recurrence, date of death, or 20 April 2023. Patients who had any stroke within 3-months were excluded. The same Cox regression analyses were used to investigate the association with stroke recurrence. As 58.7% participants (1516/2581) censored due to death, we conducted a further (Fine and Gray) competing-risks analysis using death as the competing risk event.

Generalized Estimating Equation (GEE) models binominal (link function: logit) and linear mixed regression models (LMM) (random intercept) with robust error variance were used to model the risk of self-reported stroke outcomes over 10-years post-stroke with binary (physical disability and IADL) and continuous (Physical and mental SF-12 subscales) outcomes, respectively. An exchangeable correlation structure was used for within person correlation across timepoints. Models included depression status, time in years, and their interaction. The main effect of year one difference and the time interaction over time were expressed as the differences in change in odds ratios or in mean scores for depressed vs not depressed yearly. Predicted probabilities of each binary outcome and mean score of each continuous outcome by depression status over 10-years were plotted to visualize levels. All participants with outcome data of ≥2 time-points were included in these models, and those <2 time points were classified as missing.

Weighted GEE and LMM models were performed to assess the effect of missing data (sensitivity analysis), giving each individual's data a weight inversely proportional to their probability of selection. To weight the probability of being included in the GEE and LMM, a binary indicator of completeness (0 = missing (not included in the analysis), 1 = included) was created. A logistic regression model was conducted to identify probability of being included in the analysis. Variables included in the models were age, sex, ethnicity, socioeconomic status, smoking, stroke subtype, physical disability, stroke severity and comorbidities at baseline.

All regression models were adjusted for age, sex, ethnicity, socioeconomic status, smoking, stroke subtype, physical disability, stroke severity, treatment with antidepressants and comorbidities. These covariates were chosen a priori because they are hypothesized to be confounders based on literature review and expert opinion. For the covariates with a proportion of missing data >5%, a separate category was assigned to it, for example, heart disease (No), 1 (Yes), and 2 (Unknown). Estimates conducted when the category for missing data was included were similar to those based on complete data, so the results obtained using separate categories for missing data were reported.

The same modelling strategy was used to compare 10-year outcomes in patients recovering from PSD in year 1 (depressed at 3-months, recovered at 1-year) with those never or persistently depressed. Analyses also evaluated between patients with depression symptom at 1-year or 5-years and those not depressed at these time-points.

#### Sensitivity analyses

First, as anxiety and depression scores on HADS are co-morbid,^17^ two sensitivity analyses were performed: one adjusting for anxiety scores, and the other excluding for patients with anxiety (n = 746). Second, patients who recovered at 2-years, 3-years, 4-years and 5-years after stroke were compared to those not recovered at each time-points. Moreover, in order to better elucidate the factors underlying the observed differences regarding the effect of recovery, we conducted a direct comparison between the current study to our previous one.^10^ First, we applied the statistical methods used in the previous study (logistic regression and linear regression models) to analyse data from the current paper. Additionally, we employed the current statistical method (GEE and LMM model) to analyse the data from the previous study.

All the analyses were performed using Stata 17.0 and R version 4.1.2.

### Role of the funding source

The study funders had no role in study design, data collection, data analysis, data interpretation, or the writing of the manuscript.

## Results

The SLSR recruited 7177 patients between Jan 1997 and December 2022, of whom 1495 died before the 3-month assessment. Supplementary Figure S1 shows the number of stroke survivors with depression assessment at each time-point. Among 3526 survivors who were followed-up (2156 patients lost to follow-up), 2581 (73.2%) were able to complete the HADS scale at 3 months. Of these, 918 had depression symptom (35.6%). The baseline characteristics of patients assessed for depression are presented in Table 1. Patients with depression symptom were younger, more likely to be male and black ethnicity, had more severe stroke and greater physical disabilities than those not depressed. A total of 144 (144/918, 15.7%) patients with depression symptom were taking SSRIs at 3-months after stroke, which is higher than those not depressed (98/1661, 5.9%). Comparison of the baseline characteristics between patients completing and not completing the HADS, and those who died before 3-months are presented in Supplementary Table S1. Patients completing the HADS scale had less severe strokes and lower disability at baseline.

**Table 1:** Comparison of patients’ characteristics between patients with and without depression at 3-months after stroke.

	Depressed (N = 918)	Not depressed (N = 1663)	p value
**Age (years)**	66.2 ± 14.3	67.5 ± 14.4	0.032
**Socioeconomic status (IMD)**^a^	33.9 ± 9.6	33.2 ± 9.9	0.100
**Sex (self-report)**			0.006
Male	476 (51.9)	956 (57.5)	
Female	442 (48.2)	707 (42.5)	
**Ethnicity (self-report)**			0.035
White	527 (57.4)	1041 (62.6)	
Black	318 (34.6)	503 (30.2)	
Others/Unknown	73 (8.0)	119 (7.2)	
**Stroke subtype**			0.612
Ischemic stroke	785 (85.5)	1438 (86.7)	
Haemorrhagic stroke	131 (14.3)	219 (13.2)	
Unknown	2 (0.2)	6 (0.4)	
**Smoking**			<0.0001
Never	552 (60.1)	1119 (67.3)	
Ex- smoker	293 (31.9)	472 (28.4)	
Current smoker	64 (7.0)	62 (3.7)	
Unknown	9 (1.0)	10 (0.6)	
**Physical disability**			<0.0001
Mild disability	426 (46.4)	1011 (60.8)	
Moderate to severe disability	416 (45.3)	482 (29.0)	
Unknown	76 (8.3)	170 (10.2)	
**Stroke severity**			<0.0001
Mild stroke	278 (30.3)	741 (44.6)	
Moderate to severe stroke	421 (45.9)	564 (33.9)	
Unknown	219 (23.9)	358 (21.5)	
**Comorbidities and regular medication taken at 3-months after stroke**^b^			

**Heart diseases**			0.258
No	797 (86.8)	1473 (88.6)	
Yes	118 (12.9)	188 (11.3)	
Unknown	3 (0.3)	2 (0.1)	
**Antihypertensives**			0.050
Hypertension not on medication	112 (12.2)	147 (8.8)	
Hypertension on medication	540 (58.8)	996 (59.9)	
No hypertension	265 (28.9)	518 (31.2)	
Unknown	1 (0.1)	2 (0.1)	
**Diabetes medication**			<0.0001
Diabetes not on medication	43 (4.7)	61 (3.7)	
Diabetes on medication	231 (25.2)	298 (17.9)	
No diabetes	644 (70.2)	1303 (78.4)	
Unknown	0 (0.0)	1 (0.1)	
**Antidepressants**			<0.0001
No	598 (65.1)	1284 (77.2)	
Yes	144 (15.7)	98 (5.9)	
Unknown	176 (19.2)	281 (17.0)	

a Index of multiple deprivation (IMD).

b Comorbidities at 3-months included diseases (heart disease, hypertension and diabetes) which were diagnosed at baseline or 3-months. Medication refers to regular medication taken since the diseases were diagnosed.

### Outcomes of depression at 3 months

**Fig. 1:**
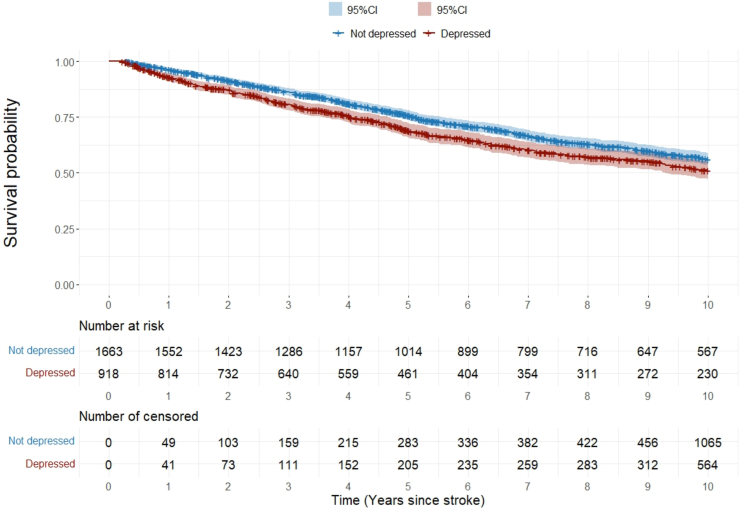
**Mortality up to 10-years after stroke by depression status at 3-months.** HR (95% CI): 1.23 (1.08–1.41); Log-rank test: p = 0.0018.

**Fig. 2:**
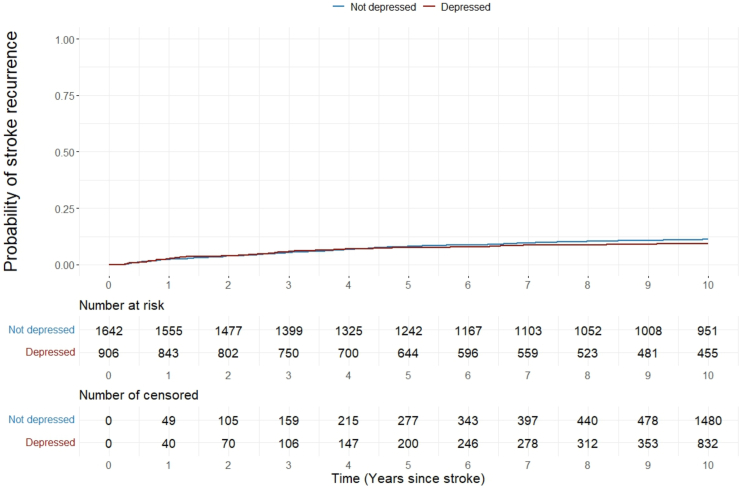
**Stroke recurrence up to 10-years after stroke by depression status at 3-months.** HR (95% CI): 0.86 (0.65–1.13); Log-rank test: p = 0.2793.

Of participants completed the HADS, 952 died over the 10-years (depressed 354 vs not depressed 598). Mortality was significantly higher in patients with depression at 3-months (Log rank test p = 0.0018; Fig. 1). After full adjustment, patients with PSD still showed higher mortality (adjusted hazard ratio (aHR): 1.18 95% CI: [1.03–1.36], p = 0.018; Table 2). Pre-stroke depression was reported in 9.9% (195/1973) of stroke survivors. The association with higher mortality did not change when patients with pre-stroke depression were excluded (Supplementary Table S2 and Supplementary Figure S2). After excluding 33 recurrences occurred within 3-months, 236 (depressed 74 vs not depressed 162) of 2548 patients experienced at least one stroke recurrence during follow-up. PSD at 3-months, was not associated with higher risks of recurrence (Log rank test p = 0.2793, aHR: 0.85 [0.64–1.14], p = 0.286) (Fig. 2 and Table 3). The competing-risks analysis confirmed the results (Supplementary Figure S3 and supplementary Table S3).

**Table 2:** Associations between depression at 3-months and mortality up to 10-years after stroke.

	aHR and 95% CI	p value
**Post-stroke depression**		0.018
No	Ref	
Yes	1.18 (1.03–1.36)	
**Age (years)**	1.07 (1.06–1.07)	<0.0001
**Socioeconomic status (IMD)**^a^	1.01 (1.01–1.02)	<0.0001
**Sex (self-report)**		0.266
Male	Ref	
Female	0.93 (0.81–1.06)	
**Ethnicity (self-report)**		<0.001
White	Ref	
Black	0.65 (0.55–0.77)	
**Stroke subtype**		0.055
Ischemic stroke	Ref	
Haemorrhagic stroke	0.80 (0.63–1.00)	
**Smoking**		
Never	Ref	
Ex-smoker	1.07 (0.92–1.24)	0.396
Current smoker	1.07 (0.67–1.70)	0.778
**Physical disability**		<0.0001
Mild disability	Ref	
Moderate to severe disability	1.55 (1.33–1.80)	
**Stroke severity**		0.046
Mild stroke	Ref	
Moderate to severe stroke	1.18 (1.00–1.40)	
**Comorbidities and regular medication taken at 3-months after stroke**		

**Heart diseases**		0.770
No	Ref	
Yes	1.03 (0.84–1.27)	
**Antihypertensives**		
Hypertension not on medication	Ref	
Hypertension on medication	0.70 (0.55–0.89)	0.004
No hypertension	0.77 (0.59–1.00)	0.050
**Diabetes medication**		
Diabetes not on medication	Ref	
Diabetes on medication	1.26 (0.87–1.83)	0.215
No diabetes	0.96 (0.67–1.36)	0.803
**Antidepressants**		0.992
No	Ref	
Yes	1.00 (0.78–1.28)	

a Index of multiple deprivation (IMD).

**Table 3:** Associations between depression at 3-months and stroke recurrence up to 10-years after stroke.

	aHR and 95% CI	p value
**Post-stroke depression**		0.286
No	Ref	
Yes	0.85 (0.64–1.14)	
**Age (years)**	1.02 (1.01–1.03)	0.001
**Socioeconomic status (IMD)**^a^	0.99 (0.97–1.00)	0.059
**Sex (self-report)**		0.501
Male	Ref	
Female	1.10 (0.84–1.43)	
**Ethnicity (self-report)**		0.001
White	Ref	
Black	1.63 (1.21–2.20)	
**Stroke subtype**		0.438
Ischemic stroke	Ref	
Haemorrhagic stroke	1.17 (0.79–1.71)	
**Smoking**		
Never	Ref	
Ex-smoker	0.91 (0.66–1.25)	0.562
Current smoker	1.68 (0.92–3.08)	0.094
**Physical disability**		0.400
Mild disability	Ref	
Moderate to severe disability	1.14 (0.84–1.57)	
**Stroke severity**		0.333
Mild stroke	Ref	
Moderate to severe stroke	0.85 (0.61–1.18)	
**Comorbidities and regular medication taken at 3-months after stroke**		

**Heart diseases**		0.821
No	Ref	
Yes	0.95 (0.2–1.45)	
**Antihypertensives**		
Hypertension not on medication	Ref	
Hypertension on medication	1.07 (0.63–3.12)	0.772
No hypertension	0.70 (0.69–3.19)	0.206
**Diabetes medication**		
Diabetes not on medication	Ref	
Diabetes on medication	1.40 (0.63–3.12)	0.405
No diabetes	1.49 (0.69–3.20)	0.308
**Antidepressants**		0.211
No	Ref	
Yes	1.33 (0.85–2.06)	

a Index of multiple deprivation (IMD).

**Fig. 3:**
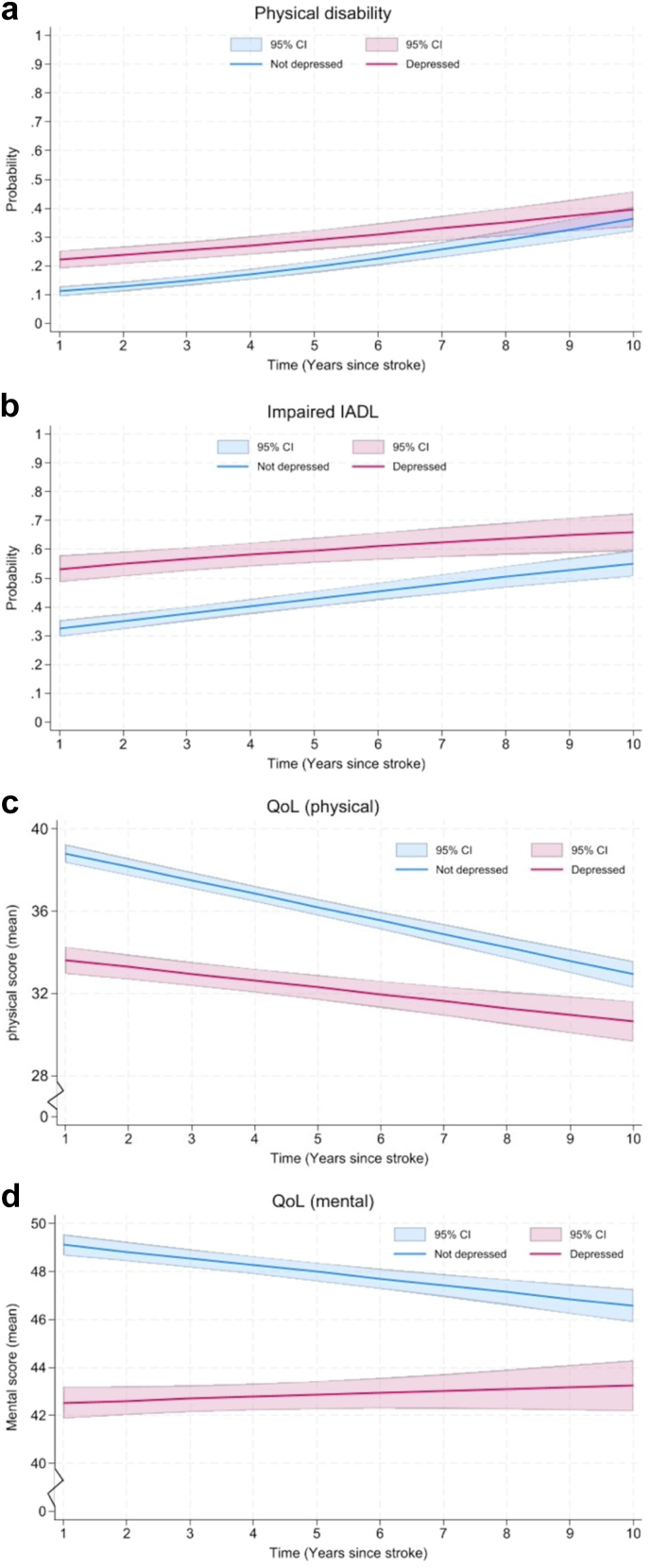
**Predicted probability and mean score of health outcomes by depression status up to 10-years after stroke.** QoL scores range from 0 to 100, with higher scores indicating better outcomes. To improve the clarity in visualizing the differences between the two groups, the y-axis representing QoL scores was segmented. a: Predicted probability of physical disability by depression status up to 10-years after stroke. b: Predicted probability of impaired IADL by depression status up to 10-years after stroke. c: Predicted mean score of QoL (physical) by depression status up to 10-years after stroke. d: Predicted mean score of QoL (mental) by depression status up to 10-years after stroke.

Among 1417 participants with ≥2 interviews during the follow-up, completion rates for different self-reported outcomes were generally over ≈90% and were similar between patients with and without depression symptom (Supplementary Table S4). PSD at 3-months was associated with higher odds of physical disability (aOR: 2.94, 95% [2.12–4.09], p < 0.0001) and lower QoL in both physical (β [95% CI], −5.93 [−7.26 to −4.60], p < 0.0001) and mental domains (−7.56 [−8.99 to −6.13], p < 0.0001) domains. These associations persisted over 10-years, though differences showed a declined trend. A significant association between PSD at 3-months and impaired IADL (2.89, [2.13–3.92], p < 0.0001) was identified, with no significant time interaction during the 10-years follow-up (Table 4 and Fig. 3). Weighted estimates confirmed the findings (Supplementary Table S5).

**Table 4:** Generalized Estimating Equation (GEE) and Linear Mixed Models (LMM) for associations between depression at 3-months and health outcomes up to 10-years after stroke.

GEE	aOR (95% CI)	p value
**Physical disability**^a^		
Depression	2.94 (2.12–4.09)	<0.0001
Depression × year	0.91 (0.87–0.97)	<0.0001
N (%)^b^	1388 (98.0)	
Follow-up years (mean ± SD)	6.1 ± 2.5	
**Impaired IADL**^c^		
Depression	2.89 (2.13–3.92)	<0.0001
Depression × year	0.96 (0.90–1.02)	0.141
N (%)^b^	1167 (82.4)	
Follow-up years (mean ± SD)	6.0 ± 2.4	

LMM	Coefficient (95% CI)	p value
**QoL (physical)**^d^		
Depression	−5.93 (−7.26 to −4.60)	<0.0001
Depression × year	0.31 (0.07–0.55)	0.011
N (%)^b^	1292 (91.2)	
Follow-up years (mean ± SD)	6.1 ± 2.4	
**QoL (mental)**^d^		
Depression	−7.56 (−8.99 to −6.13)	<0.0001
Depression × year	0.36 (0.08–0.63)	0.011
N (%)^b^	1292 (91.2)	
Follow-up years (mean ± SD)	6.1 ± 2.4	

Adjusted models included age, sex, ethnicity, socioeconomic status, smoking, stroke subtype, physical disability, stroke severity, treatment with antidepressants and comorbidities (hypertension, diabetes and heart diseases).

a Physical disability: Barthel Index <15.

b Represents the number and proportion of available samples in 1417 participants with ≥2 interviews.

c Impaired Instrumental activity of daily living (IADL): Frenchay Activities Index <15.

d Quality of life (QoL), measured by the Short Form-12 (range from 0 to 100, higher score represents better outcomes).

### Outcomes of patients depressed at 3 months not depressed at year 1 (recovered at 1-year)

Of 2581 patients assessed for depression symptom at 3-months, 1675 had complete follow-up at 1-year. Among them, 244 recovered from PSD in year 1, 897 patients did not have PSD at either time-point, and 305 exhibited depression symptom at both time-points. Compared to those not having depression symptom, recovered patients showed no differences in mortality (1.16 [0.91–1.48], p = 0.240) or stroke recurrence (0.71 [0.40–1.29], p = 0.263), but higher odds of physical disability (2.70, [1.57–4.66], p < 0.0001), impaired IADL (3.88, [2.53–5.96], p < 0.0001) and lower QoL (physical domain: (−5.78, [−7.87 to −3.69], p < 0.0001); mental domain: (−4.29, [−5.90 to −2.68], p < 0.0001) (Supplementary Figures S4 and S5 and Supplementary Tables S6 and S7).

Compared to patients with persistent PSD at 3-months and 1-year (n = 305), recovered patients (n = 244) had similar risks of mortality (1.02, [0.77–1.37], p = 0.869), but lower risks of stroke recurrence (0.47, [0.25–0.92], p = 0.026) (Supplementary Figures S4 and S5 and Supplementary Table S8). Recovery from depression within 1-year was associated with reduced physical disability (0.55, [0.36–0.85], p = 0.007), improved IADL (0.56, [0.36–0.89], p = 0.013) and better QoL in both physical (3.55, [1.30–5.80], p = 0.001) and mental domains (10.91, [8.56–13.25], p < 0.0001). Results are shown in Table 5.

**Table 5:** Comparison of health outcomes up to 10-years after stroke in patients recovering from depression at 1-year and those having persistent depression.

GEE	Depressed and recovered in year 1	
	aOR (95% CI)	p value
**Physical disability**^a^		
Depression	0.55 (0.36–0.85)	0.007
Depression × year	0.96 (0.87–1.06)	0.444
N (%)^b^	352 (96.4)	
Follow-up years (mean ± SD)	5.7 ± 2.5	
**Impaired IADL**^c^		
Depression	0.56 (0.36–0.89)	0.013
Depression × year	0.94 (0.85–1.05)	0.304
N (%)^b^	290 (79.5)	
Follow-up years (mean ± SD)	5.6 ± 2.6	

LMM	Coefficient (95% CI)	p value
**QoL (physical)**^d^		
Depression	3.55 (1.30–5.80)	0.001
Depression × year	−0.19 (−0.63 to 0.26)	0.408
N (%)^b^	329 (90.1)	
Follow-up years (mean ± SD)	5.8 ± 2.4	
**QoL (mental)**^d^		
Depression	10.91 (8.56–13.25)	<0.0001
Depression × year	−1.22 (−1.75 to −0.68)	<0.0001
N (%)^b^	329 (90.1)	
Follow-up years (mean ± SD)	5.8 ± 2.4	

Adjusted models included age, sex, ethnicity, socioeconomic status, smoking, stroke subtype, physical disability, stroke severity, treatment with antidepressants and comorbidities (hypertension, diabetes and heart diseases).

Reference group was patients having persistent depression at 3-months and 1-year.

a Physical disability: Barthel Index <15.

b Represents the number and proportion of available samples in 365 participants with ≥2 interviews.

c Impaired Instrumental activity of daily living (IADL): Frenchay Activities Index <15.

d Quality of life (QoL), measured by the Short Form-12 (range from 0 to 100, higher score represents better outcomes).

### Outcomes of depression in year 1 and year 5

2607 patients died by year 1. Among the 3391 followed-up, 2658 completed the HADS, of whom 886 (33.3%) were depressed. 4586 patients died by year 5. Of the 1908 patients who were followed-up, 1648 completed the HADS and 580 (35.2%) were depressed. Patients responded to the depression symptom assessment at 1-year or 5-years were younger and had less severe stroke and lower rates of physical disability than those not assessed (Supplementary Tables S9 and S10). Patients with PSD at 1-year presented higher mortality, but similar stroke recurrence (Supplementary Figures S6 and S7 and Supplementary Tables S11 and S12) than those not depressed. PSD at 1-year was associated with poorer functional outcomes (physical ability and IADL) and lower QoL in both physical and mental domains (Supplementary Table S13) up to 10-years after stroke. Depression symptom at 5-years after stroke was continuously associated with higher mortality, increased physical disability, reduced IADL and lower QoL up to 10-years when compared to those not depressed (Supplementary Figures S8 and S9 and Supplementary Tables S12, S14 and S15).

### Sensitivity analysis

Sensitivity analyses adjusting for anxiety slightly attenuated but did not change the association between depression and poor health outcomes (Supplementary Tables S16–S19). In analysing the effect of recovery beyond 1-year after stroke, the number of recoveries at 2-years, 3-years, 4-years and 5-years was 50,31,19,33 respectively. Recovery beyond 1-year was not associated with better health outcomes (Supplementary Tables S20 and S21). The direct comparison of current data to previous data in our previous study showed interesting findings. Contrary to the findings of our previous study, the present study showed recovery was associated with better functional ability and improved QoL when using previous paper's methods to analyse the current dataset (Supplementary Table S22). However, when current method to analyse data from the previous study, we found recovery did not alter the associates of poor health outcomes (Supplementary Table S23).

## Discussion

In this population-based study, we found that PSD was associated with higher mortality, poorer functional outcomes and lower QoL. These associations persisted up to 10-years after stroke, with the differences in physical disabilities and QoL declined and differences in IADL stabilized over time. PSD was not associated with stroke recurrence. Recovery from PSD within the first year was associated with lower risks of stroke recurrence, better functional outcomes and improved QoL in the long-term as compared to those with persistent depression symptom. Timing of PSD onset did not affect the associations with poor health outcomes.

Previous studies have reported an increased risk of mortality in patients with PSD during long follow-ups beyond 5-years. However, many of these studies employed cross-sectional designs, limiting the ability to assess the timing of depression assessments. In the present study, we demonstrate that PSD at any time-point is associated with higher mortality rates, even after making comprehensive adjustments for factors such as antidepressant treatments and comorbidities. A number of biopsychosocial mechanisms have been hypothesised to underlie the relationship between depression and mortality. First, depression is correlated with other major comorbidities, such as diabetes and hypertension. Depression may also indirectly reflect stroke severity and degree of impairments.^18^^,^^19^ Second, depression is associated with poor health behaviour, including continued higher levels of smoking and lack of adherence to medical treatments.^20^ Third, depression is associated with physiological changes, which include hyperactivity of the hypothalamic–pituitary–adrenocortical axis, changes in platelet receptors and reactivity, as well as immunological/inflammation changes, that could influence mortality.^21^ In the present study, adjustment for demographic characteristics, smoking, medical conditions and stroke severity did not explain the associations in our multivariate analyses. These findings may indirectly suggest these associations could be driven by physiological changes associated with depression or the social isolation and reduced activities, which were not controlled here.

Previous research has established that PSD is associated with poorer functional outcomes and lower QoL. However, existing evidence regarding these associations has often been cross-sectional and primarily been based on short follow-ups within 1-year. This study contributes to the literature by employing longitudinal data analysis with long-term follow-ups up to 10-years. The findings reveal a consistent association between depression symptom and poorer functional outcomes and lower QoL up to 10-years post-stroke, despite a declining trend in the associations between physical disability and QoL over time. One possible reason for the association with poor functional outcomes is that the presence of a depressed mood may lead to diminished motivation to perform rehabilitation.^22^ PSD might also influence functional outcome by decreasing physical and social function, or perhaps affecting the biological process of neuroplasticity.^23^^,^^24^ The possible overlapping between the HADS and the mental health domain of the SF-12 may partly explain the association between PSD and the lower mental health QoL.^8^ The significant time interaction implies that the effect of depression on poorer health outcomes declined over time.

Only a few studies reported the association between PSD and stroke recurrence and the results were conflicting.^25^^,^^26^ In agreement with the present study, Mei et al. found depression was not associated with stroke recurrence.^25^ In contrast, Sibolt et al. reported a higher rates of stroke recurrence in patients with PSD.^26^ Small sample (83 patients with depression), different methods in depression assessment and only included patients with ischemic stroke may explain the inconsistent results in Sibolt's study to ours.^26^ The reasons for not finding an effect on stroke recurrence in our study could be due to a lack of a true effect or inadequate statistical power to detect subtle effects (74 stroke recurrences in 916 patients).

In contrast to our earlier analysis from 2014, which showed no difference in associations,^8^ we found recovery from PSD was associated with better functional outcomes, improved QoL and lower risks of stroke recurrence. The discrepancy is attributed to the smaller sample size in the previous study, which limited the statistical power of the regression models. In our previous study, the number of cases with severe functional disabilities ranged from only 9 at 5-years post-stroke to 36 at 1-year post-stroke in patients having persistent depression symptom, a sample size too small to construct a reliable regression model. However, the reduced risks of stroke recurrence should be interpreted with caution in light of the fact that the small sample size showed limited statistical power to make a robust conclusion. It is interesting that recovery from PSD improved functional outcomes but did not reduce mortality risks. This indicates that the link between PSD and higher mortality is independent of functional impairment and more likely to be driven by physiological changes.

Recovery beyond 1-year after stroke does not appear to influence the associations with poor health outcomes, which is consistent with expectations, given that these patients exhibited persistent depression at least at two time-points, with a duration exceeding one year. However, the sample size is insufficient to develop a robust model with adequate statistical power. Further research is required to investigate how the duration of depression may impact the relationship with poor health outcomes.

PSD at 1-year or 5-years after stroke, was continuously associated with higher mortality, poorer functional outcomes and reduced QoL up to 10- years suggesting that the link between PSD and stroke outcomes does not differ depending on timing of depression onset. This implies that PSD occurring several years after stroke is similarly associated with prognosis and may be benefit from periodic clinical attention in the longer term.

Clinicians should recognize the impact of PSD on stroke survivors, not only during the immediate post-stroke period but also in the long term, once physical recovery has plateaued. Given that the resolution of depression has been shown to be associated with functional outcomes and QoL, it is recommended that targeted interventions and continuous follow-up care be implemented for patients experiencing depression to improve their long-term prognosis. Moreover, ongoing screening for PSD should be considered in the long-term, as the onset of depression several years after a stroke continues to be associated with poor health outcomes. However, the optimal screening time and frequency is inconclusive, and the optimal screening tool may vary by time since stroke. Currently, the ideal scale and whether different scales are needed at different time points poststroke is unknown. Further research is needed to define the optimal timing of screen and the best screening tool in consideration of time since stroke.

The main strengths of our study include its prospective population-based design, diverse sample, long follow-up and adjustment for a variety of confounders. The design enables us to analyse PSD as an exposure at different time-points and taken pre-stroke depression into consideration, which is reported to be associated with higher mortality.^27^ This study also has limitations. First, depression was not diagnosed with a clinical interview, as it would have been impractical to assess a large cohort of patients for such a long period of time using the DSM criteria. Although HADS is a recognized screening tool for PSD (Cronbach α >0.80; when HAD subscale scores >7 is used to identify depression; sensitivity, 0.73; specificity, 0.82),^14^ 27% of false negative errors and 18% of false positive errors is an important limitation. Thus, there is potential for misclassification with respect to gold-standard diagnostic interviews. Further studies employing clinical diagnoses of PSD are required to guide clinical recommendations. Second, the analyses could only be conducted in patients responding to the HADS. The missing data on depression symptom assessment was due to the difficulty following patients for so long and to the difficulty of some patients with severe stroke or severe cognitive impairment to respond to the HADS. The patients assessed for depression symptom had less severe strokes than those unable to respond to the HADS. Therefore, the results are likely generalizable to the mild to moderate stroke population. Third, as in almost all cohort studies, there are missing data in the follow-ups. Even though the results were similar after using inverse probability weighting to account for missing data in the outcome measurements, selection bias may still be present. Fourth, even though we have adjusted for a range of confounders that are reported to be associated with mortality, there was residual confounding between unmeasured physical, social, and behavioural factors, particularly arising from an association between stroke severity and depressive symptoms, which was not detected through the available measures. Moreover, we have dichotomised the NIHSS to measure stroke severity for clinical interpretation, which could also introduce some residual confounding. Further research is needed using mediation and causal inference techniques to explore this relationship. Fifth, the use of an assessment scale cut-off value to define depression symptom at pre-specified time points might have led to an underestimation of the prevalence of depression. Patients diagnosed in between these times and use of antidepressant medication were not used to evaluate depression symptom, which means that successfully treated depressive patients would not have been considered depressed.

### Conclusion

PSD is associated with higher mortality, poorer functional outcomes and reduced QoL up to 10-years after stroke. Recovery from depression is not associated with decreased risks of mortality but is associated with better functional outcomes and improved QoL. Timing of PSD onset does not affect the associations of poor health outcomes. These findings were limited to patients responding to the depression symptom assessment scale, who tended to have less severe stroke and may not generalize to all stroke survivors.

## Contributors

LL, MO’ and YW conceptualised the study and defined the analytical strategy; LL, XL, LDL and RP performed statistical analyses; LL drafted the manuscript; LL, IM, BA, CD, MO’ and YW review & editing the manuscript; LL, IM, BA, CD, MO’ and YW were active in funding acquisition. LL, MO’ and YW have directly accessed and verified the underlying data reported in the manuscript. All authors contributed to the discussion and interpretation of data, critically reviewed the manuscript and approved the submission of the final version. All authors had full access to all the data in the study and accept responsibility to submit for publication.

## Data sharing statement

Because of the sensitive nature of the data collected for this study, requests to access the data set for academic use should be made to the South London Stroke Register (SLSR) team: https://www.kcl.ac.uk/lsm/research/divisions/hscr/research/groups/stroke/index.aspx.

## Declaration of interests

The authors declare no conflict of interest.
