# Supplementary material for: Long-term outcomes of depression up to 10-years after stroke in the South London Stroke Register: a population-based study
Source: Lancet Reg Health Eur. 2025 May 15;54:101324. doi: 10.1016/j.lanepe.2025.101324 (PMC12145742; doi:10.1016/j.lanepe.2025.101324)
Supplement: Supplementary Materials [file mmc1.pdf]

Supplementary Table 1 Comparison of patients' baseline characteristics between patients assessed, not assessed for depression and died at 3-months after stroke.

	Assessed for depression at 3-months（N=2581）	Not assessed for depression at 3-months（N=3101）	Died at 3-months (N=1495)	P value
**Age (years)**	67.04±14.38	67.42±15.73	75.28±14.43	<0.0001
**Socioeconomic status (IMD)**[a]	33.47±9.78	33.55±9.81	35.28±9.27	<0.0001
**Sex (self-report)**				<0.0001
Male	1432(55.5)	1662(53.6)	675(45.2)	
Female	1149(44.5)	1439(46.4)	820(54.9)	
**Ethnicity (self-report)**				<0.0001
White	1568(60.8)	1759(56.7)	1062(71.0)	
Black	821(31.8)	996(32.1)	263(17.6)	
Others/ Unknown	192(7.4)	346(11.2)	170(11.4)	
**Stroke subtype**				<0.0001
Ischemic stroke	2223(86.1)	2564(82.7)	955(63.9)	
Haemorrhagic stroke	350(13.6)	499(16.1)	465(31.1)	
Unknown	8(0.3)	38(1.2)	75(5.0)	
**Physical disability**				<0.0001
Mild disability	1437(55.7)	1414(45.6)	71(4.8)	
Severe disability	898(34.8)	1307(42.2)	791(52.9)	
Unknown	246(9.5)	380(12.3)	833(42.3)	
**Stroke severity**				<0.0001
Mild stroke	1019(39.5)	1007(32.5)	88(5.9)	
Moderate and severe stroke	985(38.2)	1157(37.3)	719(48.1)	
Unknown	577(22.4)	937(30.2)	688(46.0)	

Note:
a: Index of multiple deprivation (IMD).

Supplementary Table 2 Associations between depression at 3-months and mortality up to 10-years after stroke (exclude patients with pre-stroke depression)

	aHR and 95%CI	P value
**Post-stroke depression**		0.038
No	Ref	
Yes	1.21(1.01-1.46)	
**Age (years)**	1.07(1.06-1.08)	<0.0001
**Socioeconomic status (IMD)[a]**	1.02(1.01-1.02)	<0.0001
**Sex (self-report)**		0.016
Male	Ref	
Female	0.81(0.68-0.96)	
**Ethnicity (self-report)**		<0.0001
White	Ref	
Black	0.67(0.54-0.82)	
**Stroke subtype**		0.295
Ischemic stroke	Ref	
Haemorrhagic stroke	0.86(0.64-1.14)	
**Smoking**		
Never	Ref	
Ex-smoker	0.98(0.81-1.18)	0.807
Current smoker	1.14(0.69-1.88)	0.602
**Physical disability**		<0.0001
Mild disability	Ref	
Severe disability	1.51(1.24-1.83)	
**Stroke severity**		0.215
Mild stroke	Ref	
Moderate and severe stroke	1.14(0.93-1.39)	
**Comorbidities and regular medication taken at 3-months after stroke**		
**Heart diseases**		0.667
No	Ref	
Yes	1.06(0.81-1.39)	
**Antihypertensives**		
Hypertension not on medication	Ref	
Hypertension on medication	0.83(0.62-1.10)	0.193
No hypertension	0.84(0.61-1.14)	0.264
**Diabetes medication**		
Diabetes not on medication	Ref	
Diabetes on medication	1.43(0.87-2.37)	0.162
No diabetes	1.16(0.72-1.88)	0.540
**Antidepressants**		0.216
No	Ref	
Yes	1.20(0.90-1.60)	

Note:
a: Index of multiple deprivation (IMD)

Supplementary Table 3 Associations between depression at 3-months and stroke recurrence up to 10-years after stroke (Competing risk model)

	aHR and 95%CI	P value
**Post-stroke depression**		0.280
No	Ref	
Yes	0.85(0.63-1.14)	
**Age (years)**	1.02(1.01-1.03)	0.002
**Socioeconomic status (IMD)[a]**	0.99(0.98-1.00)	0.052
**Sex (self-report)**		0.488
Male	Ref	
Female	1.10(0.85-1.42)	
**Ethnicity (self-report)**		0.002
White	Ref	
Black	1.61(1.19-2.19)	
**Stroke subtype**		0.449
Ischemic stroke	Ref	
Haemorrhagic stroke	1.17(0.78-1.74)	
**Smoking**		
Never	Ref	
Ex-smoker	0.89(0.65-1.22)	0.467
Current smoker	1.59(0.85-2.95)	0.143
**Physical disability**		0.456
Mild disability	Ref	
Severe disability	1.13(0.82-1.56)	
**Stroke severity**		0.334
Mild stroke	Ref	
Moderate and severe stroke	0.85(0.61-1.18)	
**Comorbidities and regular medication taken at 3-months after stroke**		
**Heart diseases**		0.776
No	Ref	
Yes	0.94(0.61-1.44)	
**Antihypertensives**		
Hypertension not on medication	Ref	
Hypertension on medication	1.09(0.68-1.74)	0.732
No hypertension	0.71(0.41-1.22)	0.217
**Diabetes medication**		
Diabetes not on medication	Ref	
Diabetes on medication	1.40(0.64-3.06)	0.405
No diabetes	1.49(0.71-3.16)	0.294
**Antidepressants**		0.221
No	Ref	
Yes	1.33(0.84-2.09)	

Note:
a: Index of multiple deprivation (IMD)

Supplementary Table 4 Proportions of cases and completion rates for long-term health outcomes up to 10-years after stroke by depression status at 3-months.

	Proportion of cases			Completion Rate		
	All	Depressed	Not depressed	All	Depressed	Not depressed
**Physical disability (BI<15)**						
Year 1	352/1886(18.7)	192/650(29.5) *	160/1236(12.9) *	1886/1920(98.2)	650/662(98.2)	1236/1258(98.3)
Year 2	160/935(17.1)	78/294(26.5) *	82/641(12.8) *	935/967(96.7)	294/304(96.7)	641/663(96.7)
Year 3	165/828(19.9)	85/257(33.1) *	80/571(14.0) *	828/865(95.7)	257/267(96.3)	571/598(95.5)
Year 4	139/679(20.5)	72/208(34.6) *	67/471(14.2) *	679/726(93.5)	208/227(91.6)	471/499(94.3)
Year 5	216/1031(21.0)	100/323(31.0) *	116/708(16.4) *	1031/1064(96.7)	323/331(97.6)	708/733(96.6)
Year 6	87/465(18.7)	41/130(31.5) *	46/335(13.7) *	465/485(95.9)	130/137(94.9)	335/348(96.3)
Year 7	75/381(19.7)	32/110(15.9) *	43/271(15.9) *	381/402(94.8)	110/116(94.8)	271/286(94.8)
Year 8	69/309(22.3)	26/92(28.3)	43/217(19.8)	309/328(94.2)	92/96(95.8)	217/232(93.5)
Year 9	48/232(20.7)	17/60(28.3)	31/172(18.0)	232/243(95.5)	60/64(93.8)	172/179(96.1)
Year10	46/210(21.9)	14/65(21.5)	32/145(22.1)	210/223(94.2)	65/70(92.9)	145/153(94.8)
**Impaired IADL (FAI<15)**						
Year 1	669/1525(43.9)	311/515(60.4) *	358/1010(35.5) *	1525/1920(79.4)	515/662(77.8)	1010/1258(80.3)
Year 2	511/905(56.5)	173/283(61.1) *	221/622(35.5) *	905/967(93.6)	283/304(93.1)	622/663(93.8)
Year 3	446/793(56.2)	139/245(56.7) *	208/548(38.0) *	793/865(91.7)	245/267(91.8)	548/598(91.6)
Year 4	362/652(55.5)	121/204(59.3) *	169/448(37.7) *	652/726(89.8)	204/227(89.9)	448/499(89.8)
Year 5	424/785(54.0)	143/240(59.6) *	218/545(40.0) *	785/1064(73.8)	240/331(72.5)	545/733(74.4)
Year 6	246/432(56.9)	73/122(59.8) *	113/310(36.5) *	432/485(89.1)	122/137(89.1)	310/348(89.1)
Year 7	201/355(56.7)	67/107(62.6) *	87/248(35.1) *	355/402(88.3)	107/116(92.2)	248/286(86.7)
Year 8	168/291(57.7)	47/88(53.4) *	76/203(37.4) *	291/328(88.7)	88/96(91.7)	203/232(87.5)
Year 9	120/214(56.1)	31/56(55.4) *	63/158(39.9) *	214/243(88.1)	56/64(87.5)	158/179(88.3)
Year10	80/150(53.3)	23/49(46.9)	47/101(46.5)	150/223(67.3)	49/70(70.0)	101/153(66.0)
**QoL(physical)**						
Year 1	37.64± 11.88	33.37± 10.40*	39.80± 12.00*	1608/1920(83.8)	540/662(81.6)	1068/1258(84.9)
Year 2	36.36±10.83	32.32± 9.01*	38.08± 11.08*	830/967(85.8)	248/304(81.6) *	582/663(87.8) *
Year 3	36.01±11.39	31.36± 9.48*	37.93± 11.54*	750/865(86.7)	219/267(82.0) *	531/598(88.8) *
Year 4	35.25±10.85	31.12± 8.19*	37.06± 11.38*	638/726(87.9)	195/227(85.9)	443/499(88.8)
Year 5	36.23±11.82	32.81± 10.65*	37.76± 12.01*	971/1064(91.3)	300/331(90.6)	671/733(91.5)
Year 6	35.56±11.19	31.82± 8.80*	36.93± 11.67*	421/485(86.8)	113/137(82.5)	308/348(88.5)
Year 7	36.56±11.27	32.69± 10.36*	38.09± 11.27*	338/402(84.1)	96/116(82.8)	242/286(84.6)
Year 8	35.46±10.69	31.38± 8.92*	37.08± 10.91*	289/328(88.1)	82/96(85.4)	207/232(89.2)
Year 9	34.72±10.70	30.27± 8.17*	36.37± 11.06*	207/243(85.2)	56/64(87.5)	151/179(84.4)
Year10	35.50±11.21	32.31± 9.31*	37.00± 11.73*	195/223(87.4)	62/70(88.6)	133/153(86.9)
**QoL(mental)**						
Year 1	47.26±11.24	41.78±11.85*	50.02±9.83*	1608/1920(83.8)	540/662(81.6)	1068/1258(84.9)
Year 2	46.92±10.94	41.45±11.17*	49.25±9.97*	830/967(85.8)	248/304(81.6) *	582/663(87.8) *
Year 3	46.53±11.19	41.34±12.02*	48.66±10.09*	750/865(86.7)	219/267(82.0) *	531/598(88.8) *
Year 4	46.43±11.28	41.24±11.28*	48.71±10.51*	638/726(87.9)	195/227(85.9)	443/499(88.8)
Year 5	47.05±10.93	43.48±11.74*	48.65±10.15*	971/1064(91.3)	300/331(90.6)	671/733(91.5)
Year 6	46.86±11.05	42.00±11.80*	48.65±10.21*	421/485(86.8)	113/137(82.5)	308/348(88.5)
Year 7	46.17±11.22	41.66±12.08*	47.96±10.35*	338/402(84.1)	96/116(82.8)	242/286(84.6)
Year 8	47.22±10.44	43.35±11.01*	48.76±9.82*	289/328(88.1)	82/96(85.4)	207/232(89.2)
Year 9	46.04±10.67	40.92±11.00*	47.94±9.92*	207/243(85.2)	56/64(87.5)	151/179(84.4)
Year10	46.16±10.49	43.06±10.34*	47.60±10.29*	195/223(87.4)	62/70(88.6)	133/153(86.7)

Note:
*P-value < 0.05
Proportion of cases = number of participants with poor outcome ÷ number of participants with outcome data*100%.
Completion rates= number of participants with outcome data ÷ number of participants interviewed*100%.

Supplementary Table 5 Weighted Generalized Estimating Equation (GEE) and Weighted Linear Mixed Models (LMM) for associations between depression and health Outcomes up to 10-years after stroke

Weighted GEE	aOR (95% CI)	P value
**Physical disability[a]**		
Depression	2.84(2.01-4.02)	<0.0001
Depression ×year	0.91(0.86-0.96)	0.001
**Impaired IADL[b]**		
Depression	2.85(2.05-3.98)	<0.0001
Depression ×year	0.95(0.89-1.02)	0.131
**Weighted LMM**	**Coefficient (95% CI)**	**P value**
**QoL (physical)[c]**		
Depression	-5.51(-6.40 to -4.62)	<0.0001
Depression ×year	0.32(0.17-0.47)	<0.0001
**QoL (mental)[c]**		
Depression	-6.93(-7.85 to -6.02)	<0.0001
Depression ×year	0.36(0.19-0.53)	<0.0001

Note: Adjusted models included age, sex, ethnicity, socioeconomic status, smoking, stroke subtype, physical disability, stroke severity, treatment with antidepressants and comorbidities (hypertension, diabetes and heart diseases).
a Physical disability: Barthel Index<15.
b Impaired Instrumental activity of daily living (IADL): Frenchay Activities Index <15.
c Quality of life (QoL), measured by the Short Form-12 (range from 0 to 100, higher score represents better outcomes)

Supplementary Table 6 Comparison of mortality and stroke recurrence up to 10-years after stroke in patients recovering from depression and those not having depression

Outcomes	Not depressed	Depressed and recovered in year 1	
		aHR (95%CI)	P value
Mortality	Ref	1.16(0.91-1.48)	0.240
Recurrence	Ref	0.71(0.40-1.29)[a]	0.263

Note:
Number of stroke recurrence in patients not having depression: n=75
Number of stroke recurrence in patients recovering from depression: n=14
a: Please read with caution as the sample number is not large enough to build a stable Cox regression model.
Adjusted models included age, sex, ethnicity, socioeconomic status, smoking, stroke subtype, physical disability, stroke severity, treatment with antidepressants and comorbidities (hypertension, diabetes and heart diseases).

Supplementary Table 7 Comparison of health outcomes in patients recovering from depression and those not having depression

	Depressed and recovered in year 1	
**GEE**	**aOR (95% CI)**	**P value**
**Physical disability**[a]		
Depression	2.70(1.57-4.66)	<0.0001
Depression ×year	0.89(0.82-0.97)	0.005
N(%)[b]	823(98.8)	
Follow-up years (mean± SD)	6.0±2.4	
**Impaired IADL**[c]		
Depression	3.88(2.53-5.96)	<0.0001
Depression ×year	0.91(0.83-1.00)	0.050
N(%) [b]	686(82.4)	
Follow-up years (mean± SD)	6.0±2.5	
**LMM**	**Coefficient (95% CI)**	**P value**
**QoL (physical)**[d]		
Depression	-5.78(-7.87 to -3.69)	<0.0001
Depression ×year	0.38(0.01 to 0.76)	0.044
N(%) [b]	784(94.1)	
Follow-up years (mean± SD)	6.1±2.3	
**QoL (mental)**[d]		
Depression	-4.29(-5.90 to -2.68)	<0.0001
Depression ×year	0.03(-0.30 to 0.34)	0.838
N(%) [b]	784(94.1)	
Follow-up years (mean± SD)	6.1±2.3	

Note:
Adjusted models included age, sex, ethnicity, socioeconomic status, smoking, stroke subtype, physical disability, stroke severity, treatment with antidepressants and comorbidities (hypertension, diabetes and heart diseases).
Reference group was patients not having depression at 3-months and 1-year.
a: Physical disability: Barthel Index<15.
b: Represents the number and proportion of available samples in 833 participants with ≥2 interviews.
c: Impaired Instrumental activity of daily living (IADL): Frenchay Activities Index <15.
d: Quality of life (QoL), measured by the Short Form-12 (range from 0 to 100, higher score represents better outcomes)

Supplementary Table 8 Comparison of mortality and stroke recurrence up to 10-years after stroke in patients recovering from depression and those having persistent depression

Outcomes	Depressed at both 3-months and 1-year	Depressed and recovered in year 1	
		aHR (95%CI)	P value
Mortality	Ref	1.02(0.77-1.37)	0.869
Recurrence	Ref	0.47(0.25-0.92)[a]	0.026

Note:
Number of stroke recurrence in patients having depression at 3m and 1y: n=32
Number of stroke recurrence in patients recovering from depression: n=14
Please read with caution as the sample number is not large enough to build a stable Cox regression model.
Adjusted models included age, sex, ethnicity, socioeconomic status, smoking, stroke subtype, physical disability, stroke severity, treatment with antidepressants and comorbidities (hypertension, diabetes and heart diseases).

Supplementary Table 9 Comparison of patients' characteristics between patients with and without depression at 1-year after stroke

	Depressed (N=886)	Not depressed (N=1772)	P value
**Age (years)**	65.8±14.2	66.6±14.5	0.152
**Socioeconomic status (IMD)[a]**	33.9±9.7	33.2±10.0	0.088
**Sex (self-report)**			0.018
Male	465(52.5)	1016(57.3)	
Female	421(47.5)	756(42.7)	
**Ethnicity (self-report)**			0.014
White	515(58.1)	1114(62.9)	
Black	286(32.3)	536(30.3)	
Others/ Unknown	85(9.6)	122(6.9)	
**Stroke subtype**			0.117
Ischemic stroke	745(84.1)	1539(86.9)	
Haemorrhagic stroke	138(15.6)	225(12.70)	
Unknown	3(0.3)	8(0.5)	
**Smoking**			<0.0001
Never	549(62.0)	1215(68.6)	
Ex- smoker	255(28.8)	485(27.4)	
Current smoker	68(7.7)	65(3.7)	
Unknown	14(1.6)	7(0.4)	
**Physical disability**			<0.0001
Mild disability	429(48.4)	1118(63.1)	
Severe disability	384(43.3)	487(27.5)	
Unknown	73(8.2)	167(9.4)	
**Stroke severity**			<0.0001
Mild stroke	267(30.1)	783(44.2)	
Moderate and severe stroke	391(44.1)	541(30.5)	
Unknown	228(25.7)	448(25.3)	
**Comorbidities and regular medication taken at 1-year after stroke[b]**			
**Heart diseases**			0.150
No	775(87.5)	1587(89.6)	
Yes	107(12.1)	173(9.8)	
Unknown	4(0.5)	12(0.7)	
**Antihypertensives**			<0.0001
Hypertension not on medication	184(20.8)	245(13.8)	
Hypertension on medication	502(56.7)	1093(61.7)	
No hypertension	197(22.2)	430(24.3)	
Unknown	3(0.3)	4(0.2)	
**Diabetes medication**			0.038
Diabetes not on medication	55(6.2)	80(4.5)	
Diabetes on medication	186(21.0)	318(18.0)	
No diabetes	645(72.8)	1373(77.5)	
Unknown	0(0.0)	1(0.1)	
**Antidepressants**			<0.0001
No	555(62.6)	1283(72.4)	
Yes	165(18.6)	98(5.5)	
Unknown	166(18.7)	391(22.1)	

Note:
a: Index of multiple deprivation (IMD).
b: Comorbidities at 1-year included diseases (heart disease, hypertension and diabetes) which were diagnosed at any time-points up to 1-year. Medication refers to regular medication taken since the diseases were diagnosed

Supplementary Table 10 Comparison of patients' characteristics between patients with and without depression at 5-year after stroke

	Depressed (N=580)	Not depressed (N=1068)	P value
**Age (years)**	63.1±13.2	63.4±14.0	0.723
**Socioeconomic status (IMD)[a]**	35.5±9.5	33.8±10.3	0.001
**Sex (self-report)**			0.253
Male	346(60.0)	606(56.7)	
Female	234(40.3)	462(43.3)	
**Ethnicity (self-report)**			0.157
White	345(59.5)	658(61.6)	
Black	180(31.0)	337(31.6)	
Others/ Unknown	55(9.5)	73(6.8)	
**Stroke subtype**			0.179
Ischemic stroke	484(83.5)	877(82.12)	
Haemorrhagic stroke	98(16.4)	181(17.1)	
Unknown	1(0.2)	10(0.9)	
**Smoking**			<0.0001
Never	346(60.0)	761(71.3)	
Ex- smoker	184(31.7)	255(23.9)	
Current smoker	48(8.3)	47(4.4)	
Unknown	2(0.3)	5(0.5)	
**Physical disability**			0.002
Mild disability	320(55.2)	683(64.0)	
Severe disability	182(31.4)	258(24.2)	
Unknown	78(13.5)	127(11.9)	
**Stroke severity**			<0.0001
Mild stroke	203(35.0)	428(40.1)	
Moderate and severe stroke	225(38.8)	308(28.8)	
Unknown	152(26.2)	332(31.1)	
**Comorbidities and regular medication taken at 5-years after stroke[b]**			
**Heart diseases**			0.002
No	423(72.9)	825(77.3)	
Yes	95(16.4)	111(10.4)	
Unknown	62(10.7)	132(12.4)	
**Antihypertensives**			0.043
Hypertension not on medication	80(13.8)	123(11.5)	
Hypertension on medication	247(42.6)	501(46.9)	
No hypertension	141(24.3)	284(26.6)	
Unknown	112(19.3)	160(15.0)	
**Diabetes medication**			0.041
Diabetes not on medication	21(3.6)	40(3.8)	
Diabetes on medication	119(20.5)	174(16.3)	
No diabetes	355(61.2)	725(67.9)	
Unknown	85(14.7)	129(12.1)	
**Antidepressants**			<0.0001
No	343(59.1)	686(64.2)	
Yes	104(17.9)	74(6.9)	
Unknown	133(22.9)	308(28.8)	

Note:
a: Index of multiple deprivation (IMD).
b: Comorbidities at 5-years included diseases (heart disease, hypertension and diabetes) which were diagnosed at any time-points up to 5-years. Medication refers to regular medication taken since the diseases were diagnosed

Supplementary Table 11 Associations between depression at 1-year after stroke and mortality up to 10-years after stroke

	aHR and 95%CI	P value
**Post-stroke depression at 1-year**		<0.0001
No	Ref	
Yes	1.33(1.15-1.53)	
**Age (years)**	1.08(1.07-1.09)	<0.0001
**Socioeconomic status**	1.01(1.00-1.02)	0.002
**Sex (self-report)**		0.043
Male	Ref	
Female	0.87(0.76-1.00)	
**Ethnicity (self-report)**		<0.0001
White	Ref	
Black	0.66(0.55-0.80)	
**Stroke subtype**		0.059
Ischemic stroke	Ref	
Haemorrhagic stroke	0.80(0.63-1.01)	
**Smoking**		
Never	Ref	
Ex-smoker	1.07(0.92-1.25)	0.387
Current smoker	1.29(0.80-2.08)	0.300
**Physical disability**		<0.0001
Mild disability	Ref	
Severe disability	1.42(1.22-1.65)	
**Stroke severity**		0.003
Mild stroke	Ref	
Moderate and severe stroke	1.29(1.09-1.53)	
**Comorbidities and regular medication taken**		
**Heart diseases**		0.176
No	Ref	
Yes	1.15(0.94-1.42)	
**Antihypertensives**		
Hypertension not on medication	Ref	
Hypertension on medication	0.89(0.73-1.08)	0.243
No hypertension	1.00(0.80-1.25)	0.978
**Diabetes medication**		
Diabetes not on medication	Ref	
Diabetes on medication	1.54(0.84-2.82)	0.166
No diabetes	1.42(0.97-2.08)	0.073
**Antidepressants**		0.121
No	Ref	
Yes	1.19(0.95-1.48)	

Supplementary Table 12 Associations between depression (at 1-year or 5-year) and stroke recurrence up to 10-years after stroke

	aHR (95%CI)	P value
Not depressed at 1y	Ref	
Depressed at 1y	1.16(0.77-1.75)	0.474
Not depressed at 5y	Ref	
Depressed at 5y	0.51(0.22-1.15)	0.104

Supplementary Table 13 Generalized Estimating Equation (GEE) and Linear Mixed Models (LMM) for Associations between depression at 1-year and health outcomes up to 10-years after stroke

**GEE**	**aOR (95% CI)**	**P value**
**Physical disability**[a]		
Depression	2.20(1.77-2.74)	<0.0001
Depression ×year	0.94(0.88-1.01)	0.067
N(%)[b]	1788(97.8)	
Follow-up years (mean± SD)	5.9±2.4	
**Impaired IADL**[c]		
Depression	3.00(2.22-4.06)	<0.0001
Depression ×year	1.00(0.94-1.06)	0.981
N(%)[b]	1507(82.4)	
Follow-up years (mean± SD)	5.9±2.5	
**LMM**	**Coefficient (95% CI)**	**P value**
**QoL (physical)**[d]		
Depression	-6.49(-7.60 to -5.38)	<0.0001
Depression ×year	0.16(-0.1 to 0.37)	0.152
N(%)[b]	1688(92.3)	
Follow-up years (mean± SD)	5.9±2.4	
**QoL (mental)**[d]		
Depression	-12.04(-13.25 to -10.83)	<0.0001
Depression ×year	0.99(0.73-1.24)	<0.0001
N(%)[b]	1688(92.3)	
Follow-up years (mean± SD)	5.9±2.4	

Note:
Adjusted models included age, sex, ethnicity, socioeconomic status, smoking, stroke subtype, physical disability, stroke severity, treatment with antidepressants and comorbidities (hypertension, diabetes and heart diseases).
a Physical disability: Barthel Index<15.
b: Represents the number and proportion of available samples in 1829 participants with ≥2 interviews.
c Impaired Instrumental activity of daily living (IADL): Frenchay Activities Index <15.
d Quality of life (QoL), measured by the Short Form-12 (range from 0 to 100, higher score represents better outcomes)

Supplementary Table 14 Associations between depression at 5-years after stroke and mortality up to 10-years after stroke

	aHR and 95%CI	P value
**Post-stroke depression**		0.005
No	Ref	
Yes	1.37(1.10-1.71)	
**Age (years)**	1.07(1.06-1.08)	<0.0001
**Socioeconomic status**	1.01(0.99-1.02)	0.306
**Sex (self-report)**		0.048
Male	Ref	
Female	0.80(0.64-0.99)	
**Ethnicity (self-report)**		<0.0001
White	Ref	
Black	0.53(0.40-0.72)	
**Stroke subtype**		0.851
Ischemic stroke	Ref	
Haemorrhagic stroke	1.03(0.74-1.44)	
**Smoking**		
Never	Ref	
Ex-smoker	0.93(0.72-1.20)	0.570
Current smoker	0.52(0.23-1.20)	0.124
**Physical disability**		0.369
Mild disability	Ref	
Severe disability	1.13(0.87-1.46)	
**Stroke severity**		0.157
Mild stroke	Ref	
Moderate and severe stroke	1.22(0.93-1.61)	
**Comorbidities and regular medication taken**		
**Heart diseases**		0.195
No	Ref	
Yes	1.24(0.90-1.70)	
**Antihypertensives**		
Hypertension not on medication	Ref	
Hypertension on medication	0.93(0.65-1.33)	0.685
No hypertension	1.02(0.70-1.49)	0.925
**Diabetes medication**		
Diabetes not on medication	Ref	
Diabetes on medication	1.06(0.61-1.83)	0.841
No diabetes	1.04(0.63-1.73)	0.884
**Antidepressants**		<0.0001
No	Ref	
Yes	1.83(1.32-2.54)	

Supplementary Table 15 Generalized Estimating Equation (GEE) and Linear Mixed Models (LMM) for associations between depression at 5-year and health outcomes up to 10-years after stroke

GEE	aOR (95% CI)	P value
**Physical disability**[a]		
Depression	2.42(1.39-4.22)	<0.0001
Depression ×year	1.05(0.98-1.12)	0.194
N(%)[b]	627(96.3)	
Follow-up years (mean± SD)	8.8±1.1	
**Impaired IADL**[c]		
Depression	2.69(1.76-4.11)	<0.0001
Depression ×year	1.01(0.95-1.07)	0.785
N(%) [b]	608(93.4)	
Follow-up years (mean± SD)	8.8±1.1	
**LMM**	**Coefficient (95% CI)**	**P value**
**QoL (physical)**[d]		
Depression	-6.78(-8.30 to -1.24)	0.008
Depression ×year	-0.03(-0.28 to 0.21)	0.790
N(%) [b]	589(90.5)	
Follow-up years (mean± SD)	8.8±1.1	
**QoL (mental)**[d]		
Depression	-6.76(-8.81 to -4.72)	<0.0001
Depression ×year	-0.23(-0.53 to 0.07)	0.125
N(%) [b]	589(90.5)	
Follow-up years (mean± SD)	8.8±1.1	

Note:
Adjusted models included age, sex, ethnicity, socioeconomic status, smoking, stroke subtype, physical disability, stroke severity, treatment with antidepressants and comorbidities (hypertension, diabetes and heart diseases).
a Physical disability: Barthel Index<15.
b: Represents the number and proportion of available samples in 651 participants with ≥2 interviews.
c Impaired Instrumental activity of daily living (IADL): Frenchay Activities Index <15.
d Quality of life (QoL), measured by the Short Form-12 (range from 0 to 100, higher score represents better outcomes)

Supplementary Table 16 Associations between depression at 3-months and mortality and stroke recurrence up to 10-years after stroke (further adjusting for anxiety score)

Outcomes	Not depressed	Depressed	
		aHR (95%CI)	P value
Mortality	Ref	1.18(0.99-1.39)	0.044
Stroke recurrence	Ref	0.85(0.64-1.14)	0.286

Supplementary Table 17 Associations between depression at 3-months and mortality and stroke recurrence up to 10-years after stroke (excluding patients with anxiety)

Outcomes	Not depressed	Depressed	
		aHR (95%CI)	P value
Mortality	Ref	1.24(1.03-1.50)	0.023
Stroke recurrence	Ref	0.77(0.54-1.59)	0.137

Supplementary Table 18. Generalized Estimating Equation (GEE) and Linear Mixed Models (LMM) for Associations between depression at 3-months and health outcomes up to 10-years after stroke (further adjusting for anxiety score)

GEE	aOR (95% CI)	P value
Physical disability	2.70(1.88-3.87)	<0.0001
Impaired IADL	2.28(1.73-3.01)	<0.0001
**LMM**	**Coefficient (95% CI)**	**P value**
QoL (physical)	-4.14(-5.77 to -2.52)	0.001
QoL (mental)	-4.07(-5.72 to-2.43)	<0.0001

Supplementary Table 19 Generalized Estimating Equation (GEE) and Linear Mixed Models (LMM) for Associations between depression at 3-months and health outcomes up to 10-years after stroke (excluding patients with anxiety)

GEE	aOR (95% CI)	P value
Physical disability	2.80(1.94-4.04)	<0.0001
Impaired IADL	3.12(1.88-5.17)	<0.0001
**LMM**	**Coefficient (95% CI)**	**P value**
QoL (physical)	-5.45(-7.28 to -3.61)	<0.0001
QoL (mental)	-6.21(-8.26 to -4.16)	<0.0001

Supplementary Table 20 Association between recovery at beyond 1-year and mortality and stroke recurrence

Outcomes	Mortality		Stroke recurrence	
	**aHR (95%CI)**	**P value**	**aHR (95%CI)**	**P value**
Recovered in year 2	0.74(0.41-1.31)	0.299	1.02(0.28-3.71)	0.973
Recovered in year 3	1.06(0.39-2.88)	0.910	0.16(0.01-11.78)	0.405
Recovered in year 4	0.32(0.07-1.49)	0.147	1.00(0.27-3.80)	0.992
Recovered in year 5	0.79(0.44-1.42)	0.430	2.35(0.27-20.37)	0.438

Note: Reference group was patients not recovered at each time-point.

Supplementary Table 21 Association between recovery at beyond 1-year and poor health outcomes

A: Association between recovery in year 2 and poor health outcomes

	Depressed and recovered in year 2	
**GEE**	**aOR (95% CI)**	**P value**
**Physical disability**	0.49(0.24-1.02)	0.057
**Impaired IADL**	0.26(0.10-0.65)	0.004
**LMM**	**Coefficient (95% CI)**	**P value**
**QoL (physical)**	1.61(-1.30 to 4.51)	0.278
**QoL (mental)**	5.43(3.04 to 7.82)	<0.0001

Note: reference group was patients not recovered in year 2

B: Association between recovery in year 3 and poor health outcomes

	Depressed and recovered in year 3	
**GEE**	**aOR (95% CI)**	**P value**
**Physical disability**	0.81(0.29-2.25)	0.687
**Impaired IADL**	0.50(0.18-1.36)	0.174
**LMM**	**Coefficient (95% CI)**	**P value**
**QoL (physical)**	3.54(0.53 to 6.54)	0.021
**QoL (mental)**	6.85(3.36 to 10.33)	<0.0001

Note: reference group was patients not recovered in year 3

C: Association between recovery in year 4 and poor health outcomes

	Depressed and recovered in year 4	
**GEE**	**aOR (95% CI)**	**P value**
**Physical disability**	0.33(0.12-0.97)	0.046
**Impaired IADL**	0.48(0.18-1.24)	0.129
**LMM**	**Coefficient (95% CI)**	**P value**
**QoL (physical)**	-1.54(-4.15 to 1.06)	0.246
**QoL (mental)**	6.66(2.45 to 10.89)	0.002

Note: reference group was patients not recovered in year 4

D: Association between recovery in year 5 and poor health outcomes

	Depressed and recovered in year 5	
**GEE**	**aOR (95% CI)**	**P value**
**Physical disability**	0.61(0.23-1.63)	0.323
**Impaired IADL**	0.62(0.24-1.62)	0.330
**LMM**	**Coefficient (95% CI)**	**P value**
**QoL (physical)**	4.57(1.30 to 7.84)	0.006
**QoL (mental)**	6.17(2.75 to 9.58)	<0.0001

Note: reference group was patients not recovered in year 5.

Supplementary Table 22 Comparison of health outcomes up to 5-years in patients recovering from depression and those having persistent depression (Using statistical methods in the previous paper to analyse data in the current paper)

Outcomes	Depressed and recovered in year 1	
	**Logistic regression model**	
	aHR (95% CI)	P value
**Physical disability**		
Year 1	0.44(0.27-0.69)	<0.0001
Year 5	0.34(0.16-0.72)	0.005
**Impaired IADL**		
Year 1	0.47(0.31-0.71)	<0.0001
Year 5	0.41(0.19-0.88)	0.023
	**Linear regression model**	
	Coefficient (95% CI)	P value
**QoL (physical)**		
Year 1	5.25(3.37-7.10)	<0.0001
Year 5	3.29(0.36-6.22)	0.028
**QoL (mental)**		
Year 1	12.35(10.51-14.19)	<0.0001
Year 5	3.53(0.34-6.72)	0.030

Note: reference group: patients having persistent depression at 3-months and 1-year

Supplementary Table 23 Comparison of health outcomes up to 5-years in patients recovering from depression and those having persistent depression (Using statistical methods in the current paper to analyse data in previous paper)

	Depressed and recovered in year 1	
**GEE**	**aOR (95% CI)**	**P value**
Depression	0.83(0.46-1.48)	0.523
Follow-up years (mean± SD)	3.8±1.1	
**Impaired IADL**[c]		
Depression	0.71(0.41-1.23)	0.226
Follow-up years (mean± SD)	3.9±1.1	
**LMM**	**Coefficient (95% CI)**	**P value**
**QoL (physical)**		
Depression	2.03(-0.18 to 4.23)	0.071
Follow-up years (mean± SD)	4.0±1.1	
**QoL (mental)**		
Depression	6.38(4.01 to 8.74)	<0.0001
Follow-up years (mean± SD)	4.0±1.1	

Note: reference group: patients having persistent depression at 3-months and 1-year

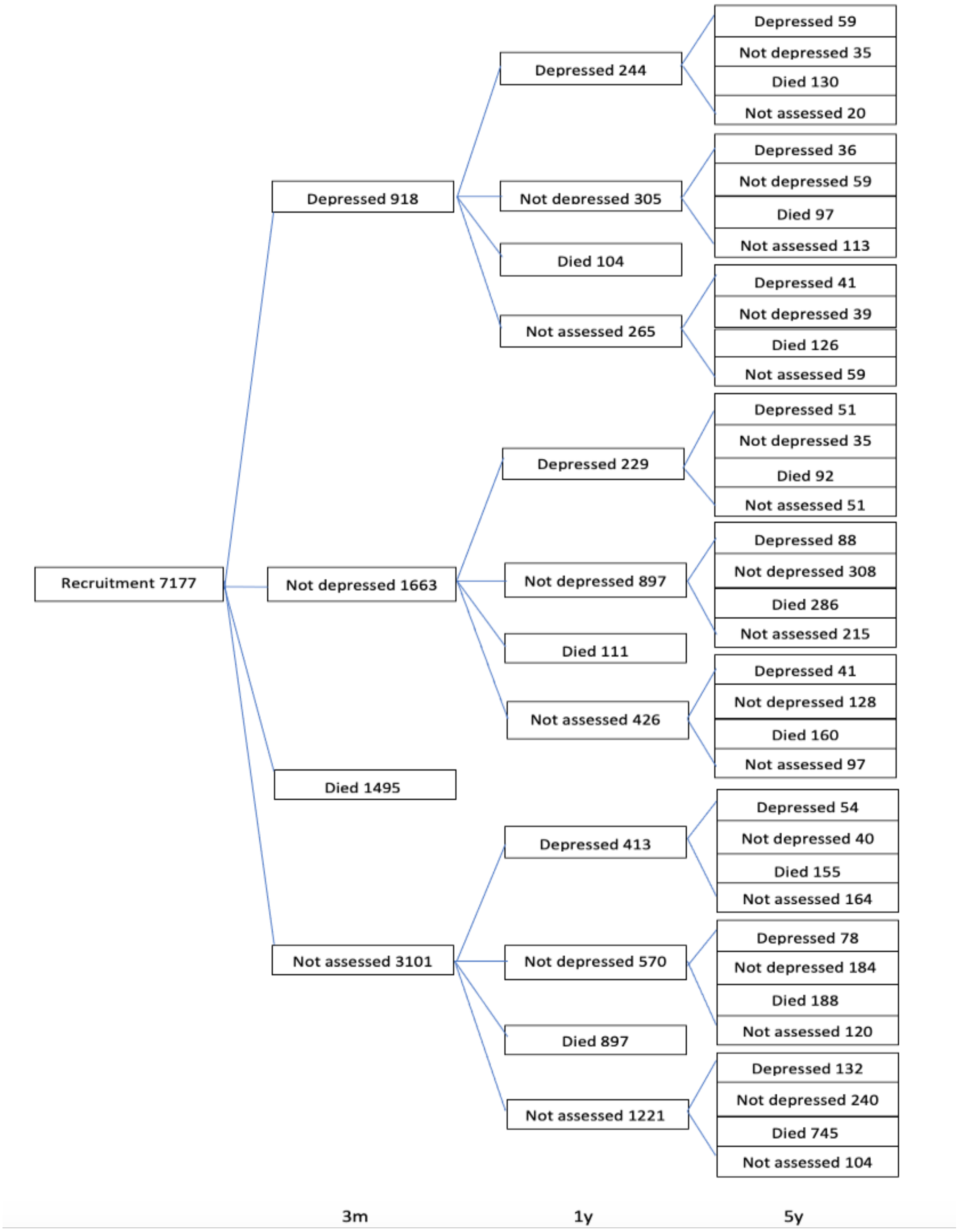

Supplementary Figure 1 Flow chart showing the number of stroke survivors with depression assessment at each time-point.

Note: There is a long duration between 1-year and 5-years follow-up points, during which depression may remit and relapse. As a result, the transitions observed between the 1-year and 5-year follow-up periods do not accurately reflect the detailed fluctuations in patients' depression status.

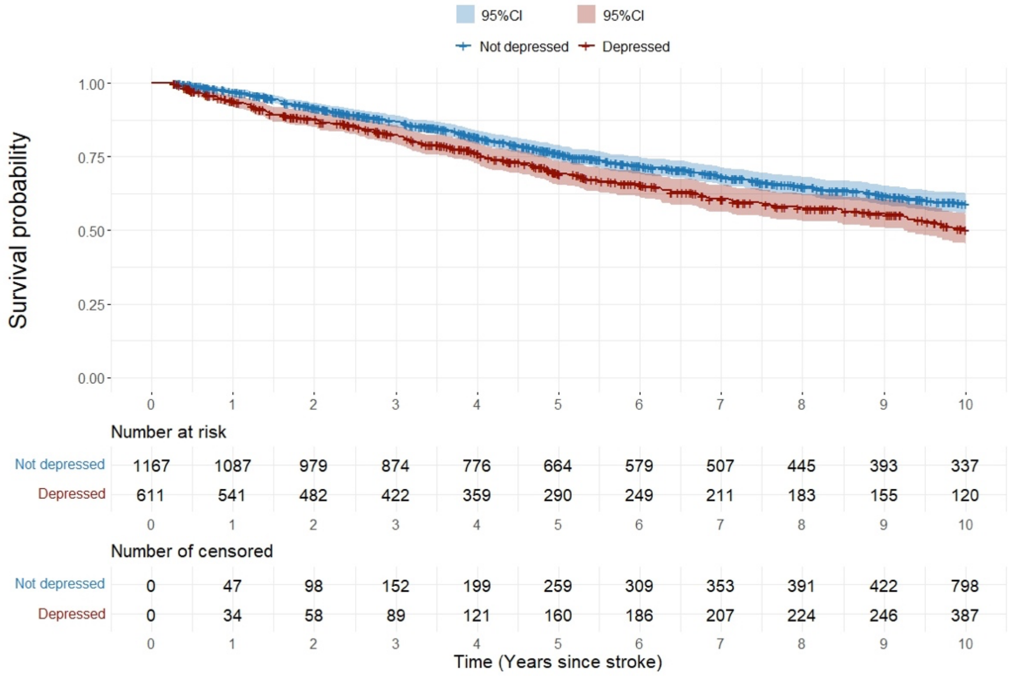

Supplementary Figure 2 Mortality up to 10-years after stroke by depression status at 3- months (exclude patients with pre-stroke depression). HR (95%CI): 1.32(1.12-1.56); Log-rank test: p=0.001

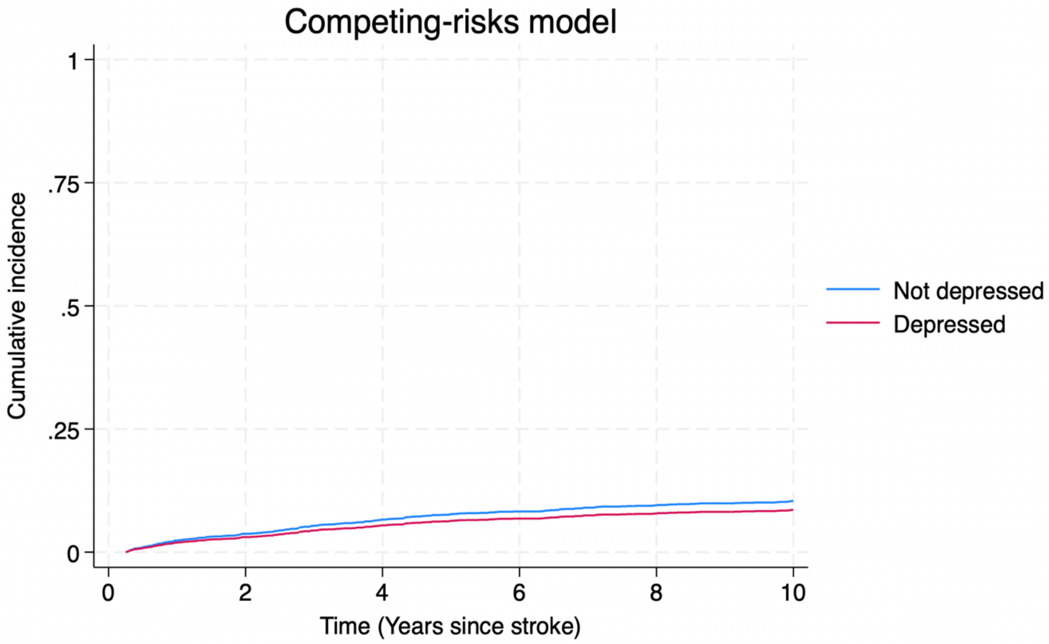

Supplementary Figure 3 Stroke recurrence up to 10-years after stroke by depression status at 3-months (competing-risk model)

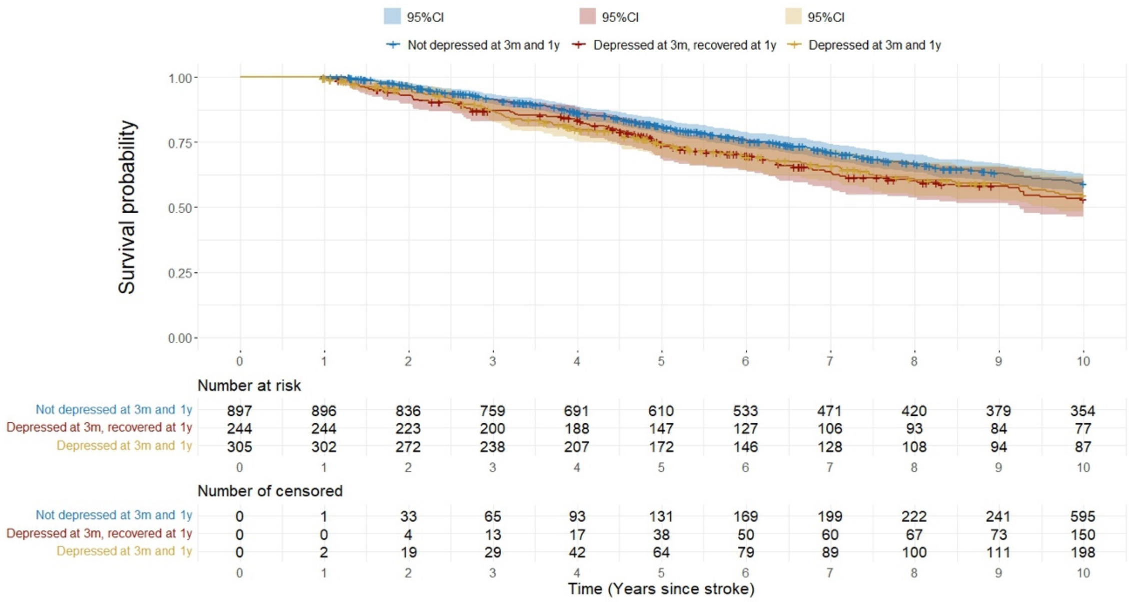

Supplementary Figure 4 10-year mortality by recovery status at 1-year. Log-rank test: P=0.0626

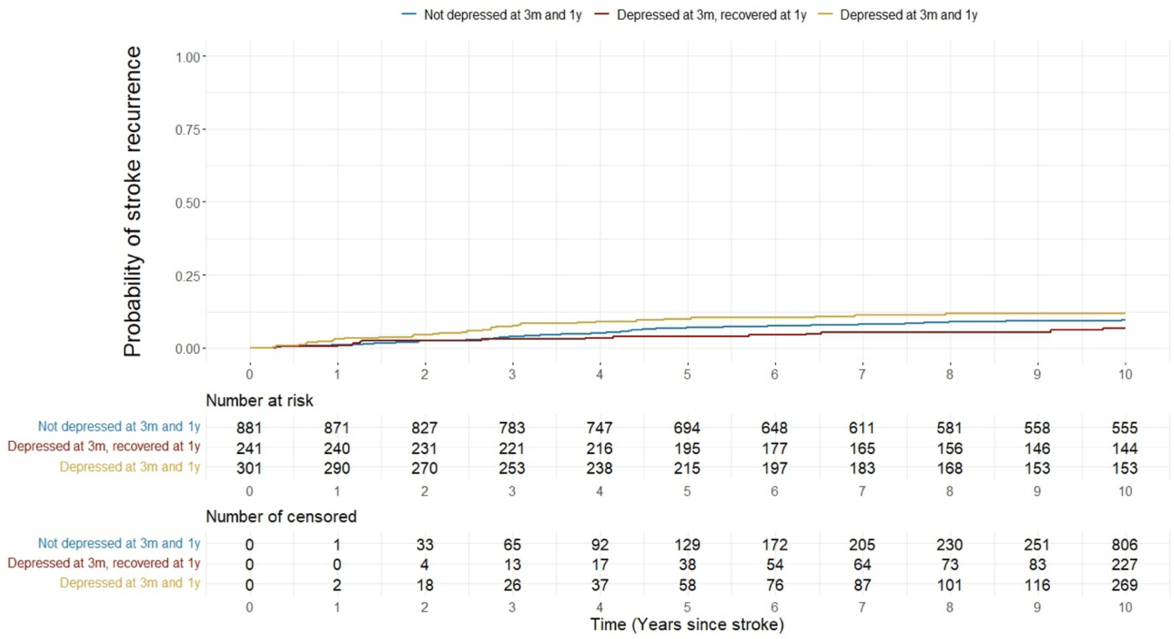

Supplementary Figure 5 10-year stroke recurrence by recovery status at 1-year. Log-rank test: P=0.0853

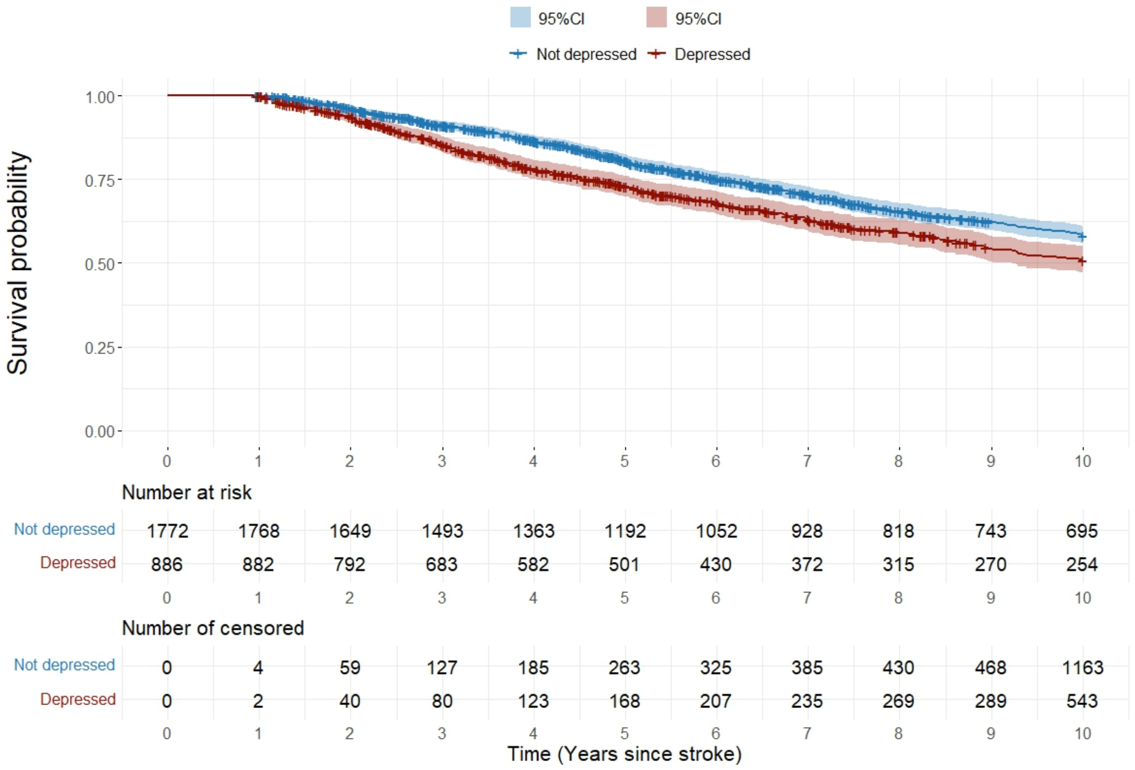

Supplementary Figure 6 Mortality up to 10-years after stroke by depression status at 1-year.
HR (95%CI): 1.32(1.16-1.51); Log-rank test: p<0.0001

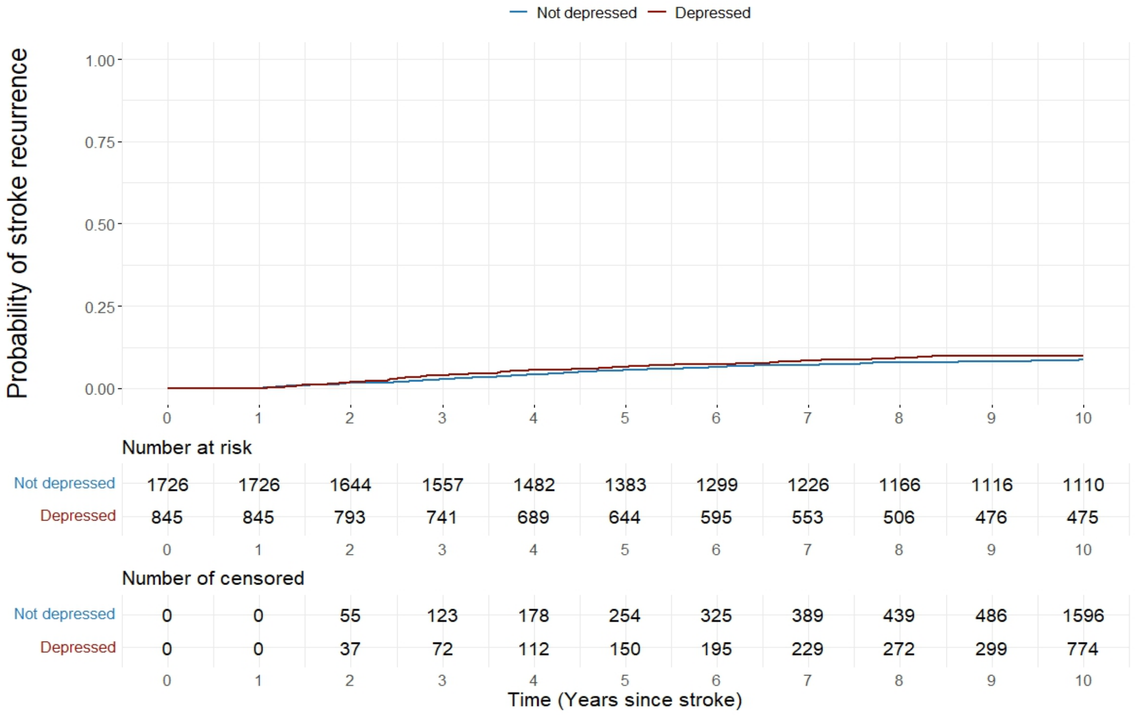

Supplementary Figure 7 Stroke recurrence up to 10-years after stroke by depression status at 1-year.
HR (95%CI): 1.17(0.88-1.56); Log-rank test: p=0.2887

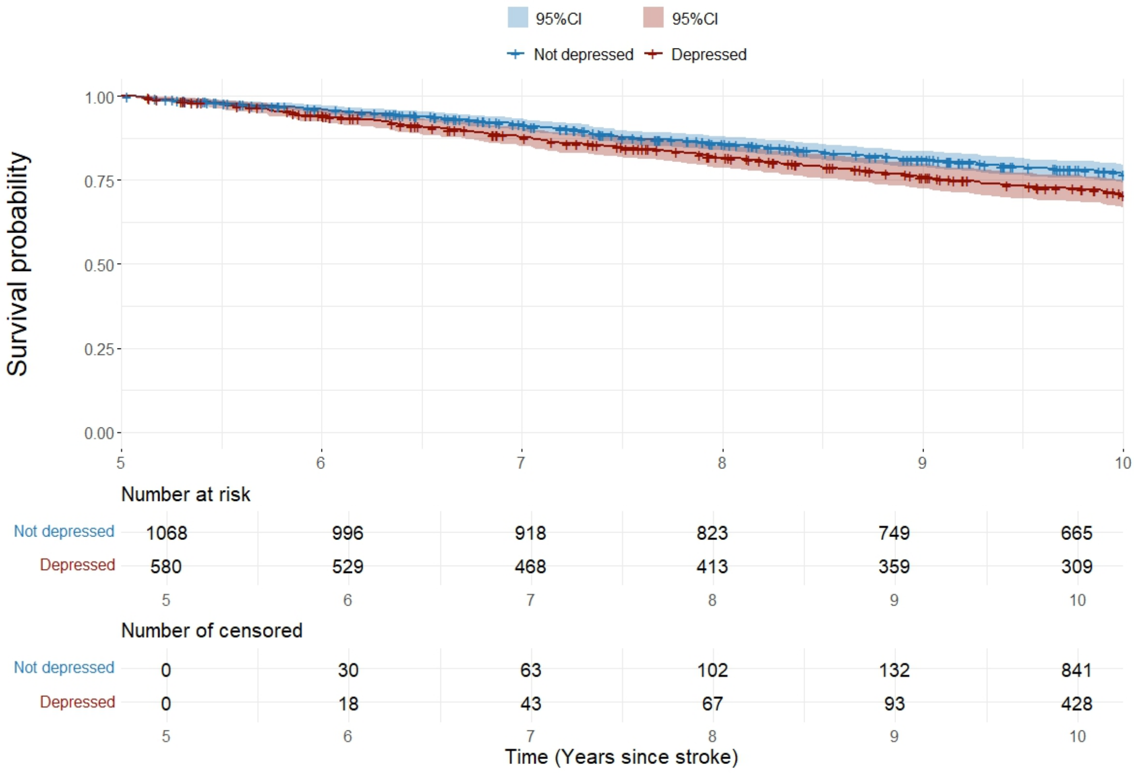

Supplementary Figure 8 Mortality up to 10-years after stroke by depression status at 5-year.
HR (95%CI): 1.32(1.07-1.62); Log rank test: p=0.0082

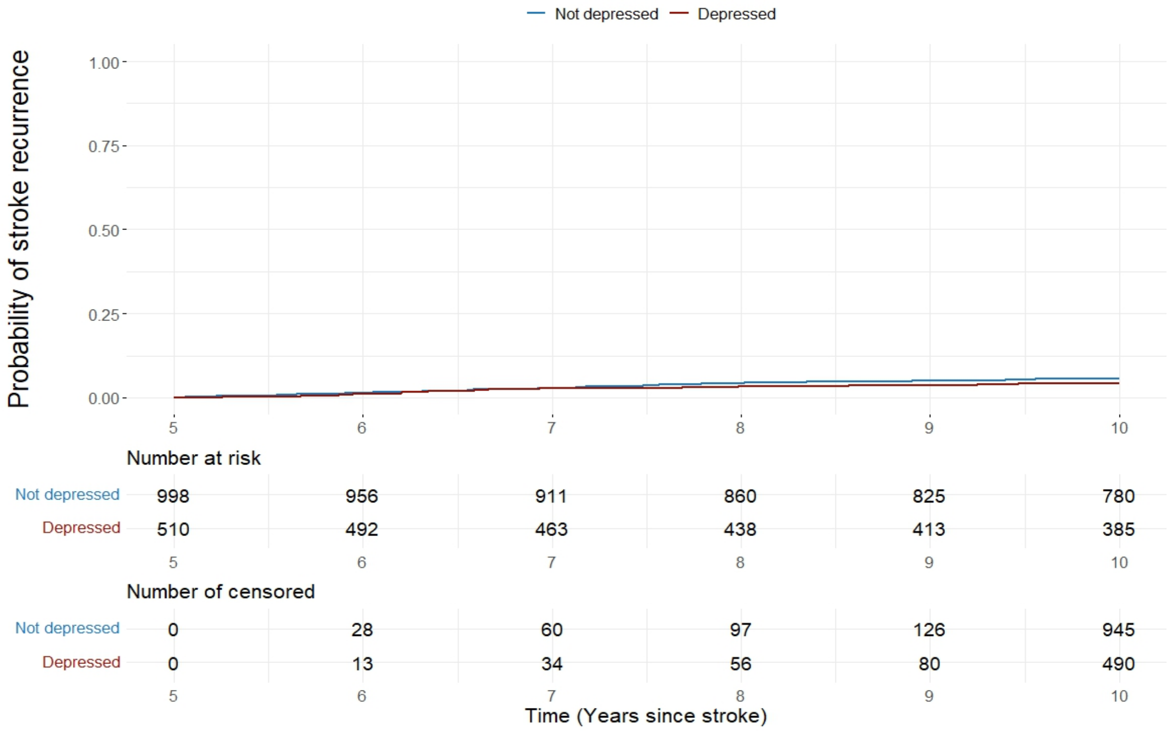

Supplementary Figure 9 Stroke recurrence up to 10-years after stroke by depression status at 5-year.
HR (95%CI): 0.74(0.44-1.24); Log-rank test: p=0.2514
